# Competitive Interactions between PIRT, the Cold Sensing Ion Channel TRPM8, and PIP_2_ Suggest a Mechanism for Regulation

**DOI:** 10.1038/s41598-019-49912-5

**Published:** 2019-10-01

**Authors:** Nicholas J. Sisco, Cole V. M. Helsell, Wade D. Van Horn

**Affiliations:** 1 School of Molecular Sciences, Arizona State University, Tempe, AZ 85287 USA; 2 The Biodesign Institute, Arizona State University, Tempe, AZ 85281 USA; 3 The Virginia G. Piper Center for Personalized Diagnostics, Arizona State University, Tempe, AZ 85281 USA; 4 The Magnetic Resonance Research Center, Arizona State University, Tempe, AZ 85287 USA

**Keywords:** Transient receptor potential channels, Ion transport, Permeation and transport, Solution-state NMR

## Abstract

TRPM8 is a member of the transient receptor potential ion channel family where it functions as a cold and pain sensor in humans and other higher organisms. Previous studies show that TRPM8 requires the signaling phosphoinositide lipid PIP_2_ to function. TRPM8 function is further regulated by other diverse mechanisms, including the small modulatory membrane protein PIRT (*p*hospho*i*nositide *r*egulator of *T*RP). Like TRPM8, PIRT also binds PIP_2_ and behavioral studies have shown that PIRT is required for normal TRPM8-mediated cold-sensing. To better understand the molecular mechanism of PIRT regulation of TRPM8, solution nuclear magnetic resonance (NMR) spectroscopy was used to assign the backbone resonances of full-length human PIRT and investigate the direct binding of PIRT to PIP_2_ and the human TRPM8 S1-S4 transmembrane domain. Microscale thermophoresis (MST) binding studies validate the NMR results and identify a competitive PIRT interaction between PIP_2_ and the TRPM8 S1-S4 domain. Computational PIP_2_ docking to a human TRPM8 comparative model was performed to help localize where PIRT may bind TRPM8. Taken together, our data suggest a mechanism where TRPM8, PIRT, and PIP_2_ form a regulatory complex and PIRT modulation of TRPM8 arises, at least in part, by regulating local concentrations of PIP_2_ accessible to TRPM8.

## Introduction

TRP ion channels are involved in diverse physiological and pathophysiological processes. Functionally, most TRP ion channels are polymodally modulated by various stimuli. TRPM8 functions in human sensory physiology as the primary cold sensor and is implicated in other physiological roles including pain, cancer, and obesity^1–5^. It is a weakly voltage sensitive, nonselective calcium-permeable cation channel that gates in response to physiologically relevant cold temperatures^6^, cooling chemicals like menthol^7^, and is sensitive to many other stimuli^8^. TRPM8 sensitivity requires the lipid phosphatidylinositol 4,5-bisphosphate (PIP_2_) to potentiate the channel for activation^9–11^ and TRPM8 function is further regulated by modulatory proteins, including the membrane protein PIRT (*p*hosphoinositide *i*nteracting *r*egulator of *T*RPs)^12–16^. PIRT modulation of TRPM8 displays an emerging trend of species-dependent functional diversity found in orthologous TRP channels^8,12^. For the mouse orthologs, PIRT increases TRPM8 sensitivity to cold, menthol, and voltage. Whereas, for the human orthologs PIRT attenuates equivalent TRPM8 mediated currents^8,12–14,17–21^.

PIRT is a phosphoinositide binding membrane protein with two transmembrane (TM) helices and intracellular N- and C-termini. It is expressed primarily in the dorsal root and trigeminal ganglia of the peripheral nervous system^22^ and appears to modulate thermosensing through apparent interactions with the cold-sensing TRPM8^12–14^ and the heat-sensing TRPV1 ion channels^12,22–24^. Additionally, PIRT‒TRPV1 interactions have also been functionally implicated in histaminergic and nonhistaminergic pruritus (itch)^25^, regulation of neuropathic pain^23,26^, and uterine contraction pain^23,25,26^. Beyond TRP channel modulation, PIRT has been implicated in modulating P2X purinoreceptors where it is coexpressed with P2X2 channels in the enteric nervous system^27^, has been reported to inhibit P2X3 currents to reduce bladder overactivity^28^, and was recently implicated in influencing metabolism in mice^29^. Despite emerging modulatory roles, little is currently known about the molecular mechanisms that underlie ion channel regulation by PIRT.

To probe the molecular mechanism of human TRPM8 (hTRPM8) modulation by PIRT, we used solution nuclear magnetic resonance (NMR) spectroscopy, microscale thermophoresis (MST), and Rosetta computational techniques to isolate how human PIRT (hPIRT), PIP_2_, and hTRPM8 interact. With solution NMR, we assigned the backbone resonances from full-length hPIRT and used solvent paramagnetic relaxation enhancement studies to experimentally identify its general membrane topology. With the assigned resonances, we used NMR-detected titrations to isolate specific hPIRT residues that bind PIP_2_ and the human TRPM8 ligand-sensing domain (hTRPM8-S1S4). Our NMR binding studies identify several hPIRT residues that bind both the hTRPM8-S1S4 and PIP_2_, which is suggestive of a competitive interaction. Using MST, we validate the NMR-detected binding studies and show that hPIRT displays classical competition with reduced affinity (i.e., a higher *K*_d_ value) for hTRPM8-S1S4 when PIP_2_ is present at saturating conditions.

To contextualize the experimental hPIRT binding results we used the recent apo avian TRPM8 cryo-EM structure^17^ to generate hTRPM8 transmembrane domain (hTRPM8-TMD, residues 672–1012) homology models using modern Rosetta techniques. We then computationally docked PIP_2_ to the hTRPM8-TMD to illuminate the potential location of the TRPM8–PIRT–PIP_2_ ternary complex. From these studies, we propose a mechanism where hPIRT exerts at least partial modulatory control of hTRPM8 by regulating PIP_2_ accessibility.

## Results

### Optimization of NMR conditions for biophysical hPIRT studies

The full-length human membrane protein PIRT was optimized for expression and purification from *E*. *coli*. This optimization yielded an average of 3 mg of purified hPIRT per liter of M9 minimal media, following a previously established protocol^12^. The purity and identity of hPIRT were confirmed by SDS-PAGE and trypsin digestion coupled with LC-MS/MS (Supplementary Fig. S1).

Selection of the hPIRT membrane mimic for NMR-based studies was made empirically by screening different detergent and bicelle conditions while monitoring ^1^H, ^15^N TROSY-HSQC NMR spectra (Supplementary Fig. S2). The optimal NMR protein spectrum typically has narrow linewidths, broad proton resonance dispersion, and unique resonances for most residues in the protein. Based on these criteria, hPIRT was eventually reconstituted in DPC (n-dodecylphosphocholine) for future studies. hPIRT is stable in DPC for weeks and gives relatively well-resolved spectra, for a helical membrane protein.

**Figure 1:**
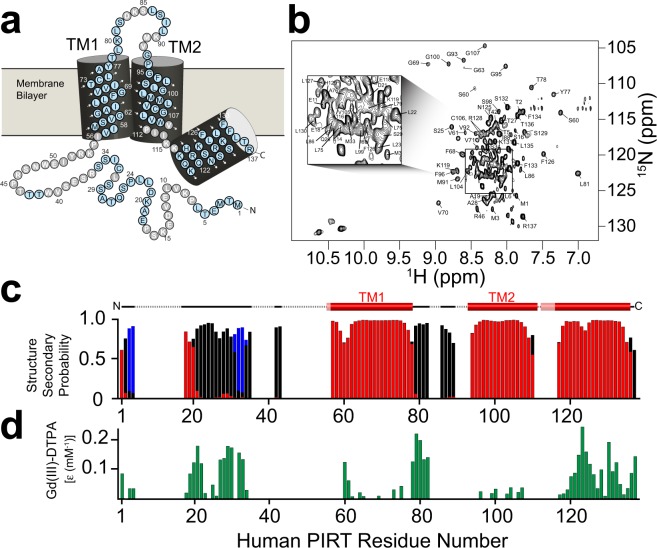
NMR derived secondary structure and topology of hPIRT. (**a**) The light blue (gray) colored circles indicate NMR assigned (unassigned) residues. (**b**) The TROSY-HSQC of hPIRT reconstituted in DPC shows a relatively well-resolved spectrum with dispersion consistent with a α-helical membrane protein. (**c**) The plot of the consensus TALOS-N predicted hPIRT secondary structure derived from experimental hPIRT Cα, C_β_, and C′ chemical shifts. Red, black, and blue bars indicate the probability of α-helix, loop, and β-sheet respectively. (**d**) Solvent paramagnetic enhancement in spin relaxation, ε, from Gd(III)-DTPA confirm the hydrophobic nature of the two transmembrane α-helices and the amphipathic nature of the C-terminal α-helix. Larger magnitude values of ε are consistent with solvent accessibility and small magnitude ε values are suggestive of protection from Gd(III)-DTPA relaxation enhancement by the membrane mimic. These results indicate that hPIRT has a relatively unstructured N-terminus, two transmembrane helices, and an amphipathic C-terminal α-helix.

**Figure 2:**
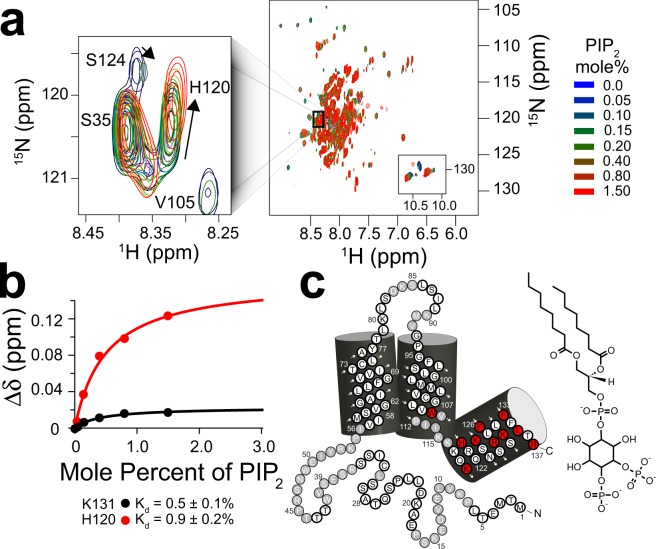
The PIRT polybasic C-terminal helix binds PIP_2_. (**a**) Overlay of eight ^15^N-TROSY HSQC hPIRT NMR spectra colored as a function of increasing PIP_2_ concentration. The left inset shows changes in H120 resonance position as the lipid is titrated. (**b**) Representative chemical shift perturbation (Δδ) titration plots for hPIRT K131 and H120 which are fit to a single binding site model as described in the methods. (**c**) hPIRT residues that specifically bind PIP_2_ are highlighted red on the topology plot.

**Figure 3:**
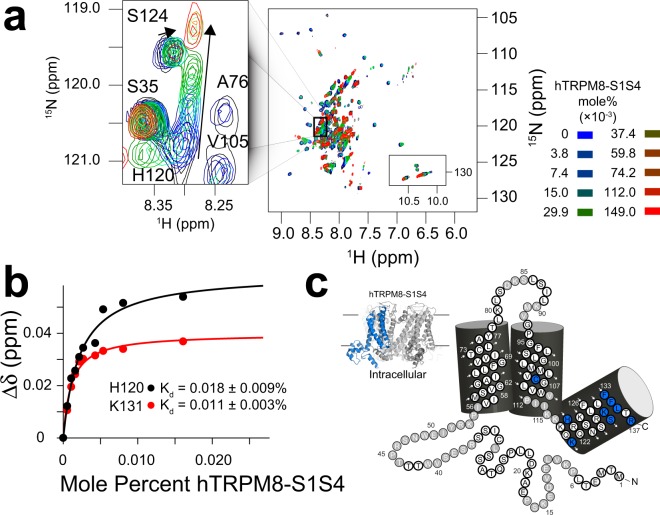
The PIRT polybasic C-terminal helix binds the hTRPM8-S1S4 domain. Uniformly ^15^N labeled hPIRT was titrated with unlabeled (^14^N, NMR silent) hTRPM8-S1S4 and monitored using ^15^N-TROSY HSQC experiments. (**a**) Overlay of ten hPIRT NMR spectra as a function of increasing hTRPM8-S1S4 concentration. At about 0.025 mol% hTRPM8-S1S4 the hPIRT H120 change in resonance position (inset) saturates while non-binding residues are generally unperturbed. (**b**) The chemical shift perturbation was quantified and fit to a standard 1:1 binding model to calculate the affinity (*K*_d_). Representative binding data for H120 and K131 are shown. (**c**) Individual hPIRT residues that specifically bind to the hTRPM8-S1S4 are mapped onto the hPIRT topology diagram. The hTRPM8-S1S4 is highlighted blue next to the hPIRT topology to emphasize the domain being titrated. These NMR data agree with a previously published study^12^.

Far-UV CD and NMR binding studies were performed to validate the suitability of DPC as a membrane mimic. Previous bioinformatic analysis of hPIRT predicts a helical two-span membrane protein^22^. The far-UV CD spectrum of DPC reconstituted hPIRT (Supplementary Fig. S1) is consistent with an α-helical protein, with spectral minima near 208 nm and 222 nm and a positive maximum near 193 nm. NMR-derived secondary structure analyses (described in detail below) are also consistent with a two-span helical membrane protein (Fig. 1). NMR-detected titrations of hPIRT in DPC micelles with PIP_2_ and hTRPM8 S1–S4 show saturable binding isotherms (Figs 2 and 3), a hallmark of specific and direct binding. These results indicate that expression and reconstitution of hPIRT in DPC retains a biologically relevant structural conformation; the significance of the affinities are discussed below.

### Human PIRT backbone NMR resonance assignment and secondary structure prediction

TROSY 3D NMR experiments were used to assign 68.6% (Fig. 1a) of the hPIRT backbone resonances (^15^N, ^1^H, ^13^C_β_, ^13^C_α_, and ^13^C′) from a U-^13^C/^15^N labeled hPIRT sample in DPC micelles at 40 °C and pH 6.5. Representative assignment data are shown in Supplementary Fig. S3. The resonance assignments (Fig. 1b) were used as input for TALOS-N^30,31^, from which secondary structure elements of hPIRT were predicted (Fig. 1c). Paramagnetic relaxation enhancement studies of hPIRT with Gd(III)-DTPA, a hydrophilic paramagnetic probe, were used to experimentally map the hPIRT membrane topology (Fig. 1d)^32,33^. The data suggest that the start of TM1 is likely near Pro55 and ends at Ala76. TM2 likely begins at Gly93; however, the C-terminal end of TM2 is not clearly defined in our data. Nonetheless, PsiPred-based bioinformatics predictions suggest that TM2 terminates between Pro112 and Lys115^34^. After TM2, the remaining C-terminal residues comprise an amphipathic α-helix (Asp125 to Arg137), as indicated by NMR with the Gd(III)-DTPA paramagnetic probe studies (Fig. 1d). The amphipathic C-terminal helix provides a region that can hug the inner membrane leaflet and allow for interactions at the bilayer interface with lipids or proteins.

Most of the resonance assignments come from the transmembrane helices and the putative C-terminal PIP_2_ binding pocket, which are DPC-associated and typically less dynamic than loops and soluble regions. Predictions from PsiPred suggest that the hPIRT N-terminus is disordered, which is consistent with the observed NMR spectral overlap that hinders resonance assignment^34,35^. Further complicating NMR assignment of the N-terminus is that this region includes several proline residues (Pro7, Pro16, Pro23, Pro45, and Pro55) and there are several repeated amino acid stretches (Leu22-Leu23, Ser29-Ser30, Ser34-Ser35, and Thr42-Thr43-Thr44) making the assignment of the N-terminus difficult. Omitting the hPIRT N-terminus and extracellular loop regions, the remainder of the protein is assigned to about 89%. Thus, the putative C-terminal PIP_2_ binding pocket and the majority of the transmembrane helices have been successfully assigned.

### NMR-detected human PIRT titrations with PIP_2_ and the hTRPM8-S1S4 domain

NMR assignment allows for identification of residue-specific information in NMR-detected binding studies. Thus these studies provide affinity information, identify regions involved in binding, and provide insight into the binding modalities. Saturation of chemical shift perturbation (Δδ) is indicative of specific and direct binding^36^. NMR-based hPIRT-detected titrations of PIP_2_ (Fig. 2) and hTRPM8-S1S4 (Fig. 3) identify several hPIRT residues with saturable binding curves.

In membrane protein binding studies, where a ligand strongly partitions into the membrane or membrane mimic, dissociation constants in units of mole percent better reflect the physical constraints of the interaction^37,38^. Previous studies have estimated that the physiological concentration of PIP_2_ in the inner leaflet of dorsal root ganglia (DRG) is ca. 1 percent of membrane mass^39^. NMR-detected hPIRT binding studies of PIP_2_ show saturable chemical shift perturbation (Fig. 2a,b) for select residues in the intracellular side of TM2 and the C-terminal amphipathic helix (Fig. 2c). The hPIRT affinity for PIP_2_ (Supplementary Table S2) is in the range of the physiological PIP_2_ concentration, suggesting a biologically relevant interaction.

We have previously shown that hPIRT directly binds the hTRPM8-S1S4 domain with a stoichiometry of ~1:1^12^. Here we use units of mole percent and hPIRT resonance assignments to further interrogate the hPIRT–hTRPM8-S1S4 interaction (Fig. 3a). From these studies, we identify many hPIRT residues that bind specifically to the hTRPM8-S1S4 domain (Fig. 3b,c).

### MST Binding measurements and competition assay

**Figure 4:**
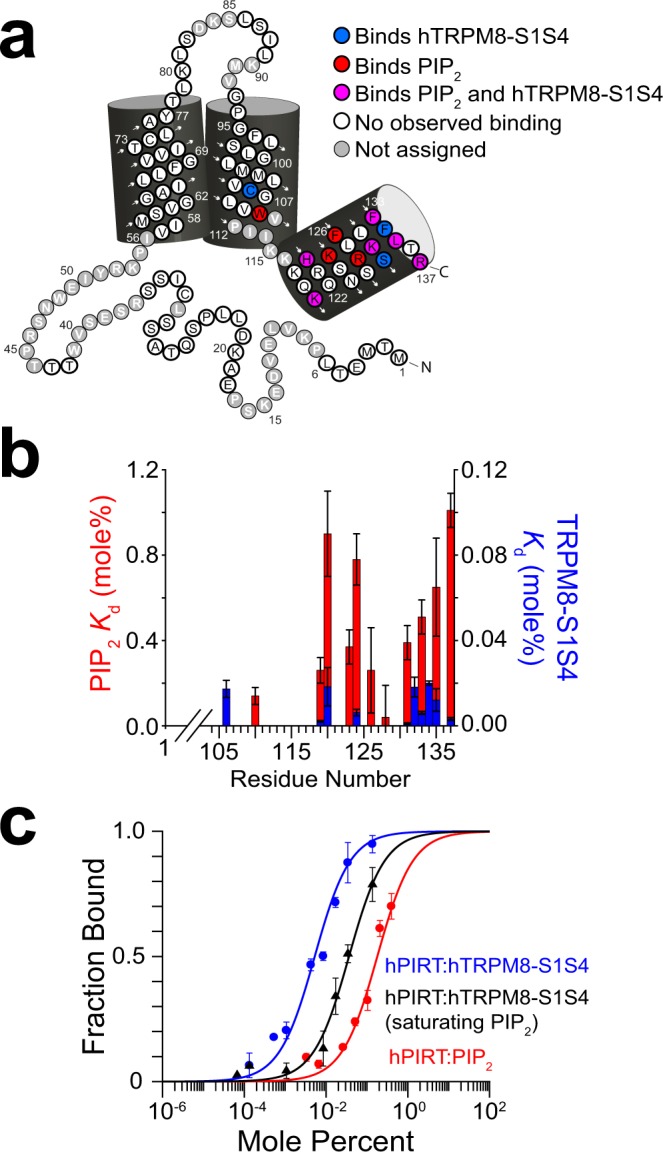
Competitive PIRT binding between PIP_2_ and TRPM8-S1S4. (**a**) NMR-detected titrations show hPIRT residues that bind both to PIP_2_ and hTRPM8 (magenta) are generally localized to the intracellular amphipathic helix. Residues that exclusively bind PIP_2_ (hTRPM8-S1S4) are colored red (blue). The overlapping binding regions indicate a competitive interaction for hPIRT. (**b**) Comparative binding affinities between PIP_2_ and the hTRPM8-S1S4 domain to hPIRT show overlapping binding sites and a higher hPIRT affinity for hTRPM8-S1S4. (**c**) MST measurements highlight the apparent competitive binding relationship between hPIRT, hTRPM8-S1S4, and PIP_2_ suggested by the NMR data. When hPIRT is titrated with the TRPM8-S1S4 domain in the presence of saturating PIP_2_, the affinity curve shifts rightward, indicative of competition. MST *K*_d_ values are 5.3 ± 0.4, 36.5 ± 0.5, and 180 ± 60 millimole percent for hPIRT:hTRPM8-S1S4, hPIRT:hTRPM8-S1S4 + PIP_2_, and hPIRT:PIP_2_ respectively.

The NMR studies identify several hPIRT residues involved in binding the hTRPM8-S1S4 domain, including Lys119 and His120, that overlap with the putative PIP_2_ binding pocket (Figs 2c, 3c and 4a) suggesting the potential for a competitive interaction (Supplementary Table S2). We used MST to interrogate the apparent hPIRT competitive binding for PIP_2_ and the hTRPM8-S1S4. In Fig. 4a, the overlapping binding residues are mapped onto the hPIRT topology, which suggests that the C-terminal helix is predominantly responsible for binding to both the hTRPM8-S1S4 and PIP_2_, with relative affinities suggesting hPIRT has a higher affinity for hTRPM8-S1S4 than PIP_2_ (Fig. 4b). MST has been used previously to measure affinities for hTRPM8-S1S4 ligand interactions^37^, PIP_2_ protein interactions^40^, and is highly sensitive to minor differences in binding^41^. *K*_d_ values obtained from MST measurements of the affinities of hPIRT for PIP_2_ and hTRPM8-S1S4 are 0.18 ± 0.06 mole% and 0.0053 ± 0.0004 mole%, respectively (Fig. 4c) and corroborate the NMR determined affinities (Supplementary Table S2). Competitive hPIRT binding to hTRPM8-S1S4 in the presence of a saturating PIP_2_ concentration shows a seven-fold decrease in affinity to 0.0365 ± 0.0005 mole% (Fig. 4c). A negative control for the MST studies shows that hPIRT does not bind to DMSO (Supplementary Fig. S4).

### Rosetta comparative modeling of human TRPM8 transmembrane domain (TMD)

RosettaCM was used to generate a hTRPM8-TMD homology model to integrate previously published information about hTRPM8, PIP_2_, and hPIRT. Previous biochemistry and functional studies have identified regions of hTRPM8 that have been linked to PIP_2_ regulation^9–11^. Previous biophysical and cellular studies have suggested that hPIRT interacts with both the hTRPM8-S1S4 and pore domains^12^. The hTRPM8 TMD decoys were built into the electron density map from the avian *Ficedula albicollis* TRPM8 (*Fa*TRPM8, 6BPQ) cryo-EM structure^17^ using RosettaCM electron density tools^42,43^, and TRPM4 as templates (Supplementary Fig. S5). Implicit membrane potentials^44–46^ were also used and resulted in models of human TRPM8 that include loops and side chains that are absent in the apo *Fa*TRPM8 structure (Fig. S6a). The output shows a characteristic energy funnel of decreasing energy scores as a function of the RMSD that is typically consistent with sampling convergence (Fig. S6). Refinement of the two most populated clusters gives way to high-quality homology models as judged by analysis of the centroid model from the top ten final decoys, which has an EMRinger score of 1.6 and MolProbity clash score of 4.99 (94^th^ percentile) after accounting for the number of residues^47,48^. The resulting hTRPM8 TMD homology models also fit well to the *Fa*TRPM8 electron density (Fig. S6a). Analysis of the top ten final models also shows that there is relatively little Cα deviation especially in the transmembrane regions (Fig. S6a), which are those that have been implicated in hPIRT modulation^12^. The hTRPM8-TMD models contain Lys680, Arg688, Arg851, Lys995, Arg998, and Arg1008 (Fig. S9) that were previously suggested to interact with the electronegative phosphoryl groups in PIP_2_^9,17^ but were unresolved in the apo *Fa*TRPM8 structure.

### Computational PIP_2_ docking to the hTRPM8–TMD

**Figure 5:**
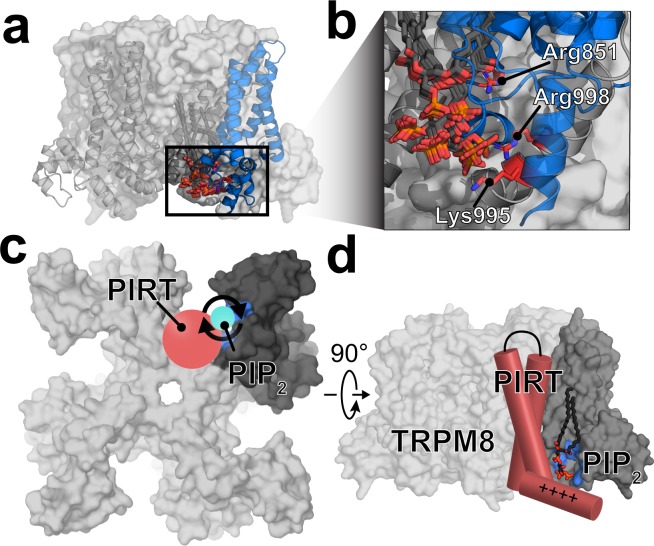
Computational docking was used to identify the PIP_2_ binding site of hTRPM8 and suggests the region where PIRT binds TRPM8. (**a**) The largest clusters of docked PIP_2_ are localized around the positively charged intracellular space near the TRP domain interfacing with the S1S4 domain. (**b**) Arg851, Lys995, and Arg998 in TRPM8 were previously identified as PIP_2_ sensitive in early cellular studies and are shown near the docked PIP_2_. The results from the hPIRT binding and lipid docking studies implicate the binding site for PIRT modulation of TRPM8. (**c**) A proposed mechanism of PIRT regulation emerges where PIP_2_ accessibility for TRPM8 activation is regulated by PIRT binding leading to channel regulation. PIRT is shown in red, PIP_2_ in cyan, the TRPM8-S1S4 domain in dark grey, and Arg851, Lys995, and Arg998 are highlighted in a blue surface for a monomer. (**d**) Cartoon of PIRT, near the PIP_2_ binding site of TRPM8. This interaction is consistent with PIRT binding both the TRPM8-S1S4 domain (dark grey) and PIP_2_ competitively and places PIRT near the pore domain which has been shown to be important in PIRT modulation of TRPM8 previously. For simplicity, the figure only depicts a single binding interaction, previous studies suggest that there are up to four PIRT binding sites in a tetrameric TRPM8 channel.

Rosetta-based ligand docking was used to probe the PIP_2_ binding site of hTRPM8. Unrestrained docking of PIP_2_ to the hTRPM8-TMD was used to generate 10,000 TRPM8–PIP_2_ decoys. These docked complexes identify a PIP_2_ binding pocket that is consistent with PIP_2_-dependent TRPM8 residues that were previously determined experimentally (Fig. S7)^9–11^. Generally, the unrestrained PIP_2_ docking appears to highlight a positively charged area near the juxtamembrane region at the nexus of the hTRPM8 pore, S1-S4, and TRP domains. To refine the docking with functional-based restraints, we selected centroids from the largest PIP_2_ binding clusters (Supplementary Figs S8 and S9) to seed the next rounds of docking using the Rosetta Matcher constraint protocols^49^. Experimentally, residues Lys995, Arg998, and Arg1008 have been implicated in PIP_2_ regulation of TRPM8^9^. However, because Arg1008 is not spatially close to Lys995 nor Arg998, we performed two additional rounds of docking using constraints to guide PIP_2_ near Lys995/Arg998 (Fig. S7c,d) or Arg1008. With 1000 experimentally guided decoys, seeded from the 10,000 unrestrained decoys, PIP_2_ binding near hTRPM8 residues Lys995 and Arg998 were consistently identified in both the unrestrained (Supplementary Fig. S9) and Lys995/Arg998 restrained docking (Fig. 5). Decoys with PIP_2_ in the vicinity of Arg1008 were not populated in the large, unrestrained docking of 10,000 PIP_2_ decoys, which is in agreement with recent PIP_2_ bound structures^50^. Nonetheless, in a subsequent docking study, PIP_2_ was guided near Arg1008; however, the output from these 1000 decoys and an additional 3000 decoys did not show convergence (Supplementary Fig. S10). Generally, the Arg1008 guided decoys did not result in a consensus PIP_2_ binding site. During the manuscript review process, additional structures of *Fa*TRPM8 were determined using cyro-EM^50^. Two of these structures, namely 6nr2 and 6nr3, identify the binding site of PIP_2_ in TRPM8. These structures are remarkably consistent with the docking outcomes as shown in Fig. 5 and validate the location of the PIP_2_ binding site. Comparison of the cryo-EM structures with the computational docking are shown in and compared in Fig. S11. Given that hPIRT binds PIP_2_ and the hTRPM8-S1S4 domain, and recent structural data that validate our comptuationally identified TRPM8–PIP_2_ binding site that is centered around the TRPM8-S1S4 domain, it follows that PIRT presumably binds TRPM8 near the TRPM8–PIP_2_ binding site to excert its modulatory impact.

## Discussion

Since its identification in 2008, PIRT has been implicated in the modulation of TRPM8, TRPV1, P2X2, and P2X3 ion channels with corresponding functional consequences that impact hot and cold temperature sensing^12,14,22^, neuropathic^23^ and uterine contraction pain sensing^26^, histaminergic and non-histaminergic itch^25^, enteric nervous system regulation^27^, and bladder overactivity^28^. Initial murine studies of PIRT modulation of TRPM8 functionally implicated the TRPM8–PIRT complex in thermosensing and showed that PIRT increases TRPM8 channel conductance^13,14^. Recent cellular studies identified species-dependent effects confirming that mouse PIRT enhances mouse TRPM8 currents while human PIRT attenuates hTRPM8 conductance^12^. As part of these cellular studies, hPIRT was shown to directly bind to the hTRPM8-S1S4, and the hTRPM8 pore domain was identified as central to the species-dependent functional effects^12^. To expand our understanding of hTRPM8 modulation by hPIRT at the molecular level, we experimentally determined the hPIRT membrane topology and characterized the direct binding of hPIRT to both PIP_2_ and the hTRPM8-S1S4 domain using solution NMR. These NMR-detected binding data indicate a competitive interaction between hPIRT binding PIP_2_ and the hTRPM8-S1S4. MST studies of hPIRT binding further confirm a competitive interaction between PIP_2_ and hTRPM8-S1S4. To put our experimental results in a molecular context, we used computational techniques to construct a homology model of hTRPM8-TMD guided by the electron microscopy density map from the avian *Fa*TRPM8 structure^17^ and the homologous mouse TRPM4 structure^51,52^. Using this homology model, we used experimentally guided computational docking of PIP_2_ to identify the binding site^9^. Given that hPIRT binds the hTRPM8-S1S4 and PIP_2_, and hTRPM8 binds PIP_2_, these computational studies allow us to predict the general region where hPIRT binds hTRPM8 (Fig. 5c,d). Our studies herein experimentally identify the hPIRT topology, help illuminate the mechanism for hPIRT modulation of hTRPM8, and provide insight into where hPIRT potentially binds hTRPM8.

MST binding studies confirm results from NMR showing hPIRT interacts with both PIP_2_ and the hTRPM8-S1S4 domain (Fig. 4) and that hPIRT affinity for the hTRPM8-S1S4 domain that is about 35-fold higher (lower *K*_d_ value) than that for the PIP_2_ lipid. While the MST and NMR binding data are generally in good agreement, the relatively small differences in the affinities observed here are likely explained by the fact that the NMR experiments were carried out near physiological temperature (40 °C) and the MST measurements at room temperature (25 °C). The MST competitive binding assay monitoring hTRPM8-S1S4 affinity in the presence of saturating PIP_2_ supports the overlapping hPIRT binding sites identified by NMR. Specifically, in the presence of saturating PIP_2_, the affinity between hPIRT and hTRPM8-S1S4 is decreased by ca. 7-fold as noted by increased *K*_d_ values (Fig. 4b). Taken together, the observation from NMR studies that identify overlapping hPIRT binding residues for PIP_2_ and the hTRPM8-S1S4 domain combined with the MST data showing a decrease in affinity for the hTRPM8-S1S4 domain in the presence of PIP_2_ is indicative of a competitive interaction. In the context of functional regulation, we suggest that the mechanism for hPIRT regulation of hTRPM8 relies at least in part on modulating channel access to PIP_2_, a required lipid cofactor for hTRPM8 function^9^.

A detailed structural understanding of the TRPM8–PIP_2_ complex would help explain how PIRT modulates TRPM8 function and identify potential modes of interaction between hTRPM8 and hPIRT. During the manuscript peer-review process, two *Fa*TRPM8 cryo-EM structures were determined with PIP_2_ bound and resolved^50^. These PIP_2_ bound TRPM8 structures, validate the Rosetta-based PIP_2_ docking to hTRPM8 (Figs 5 and S11). Our docked hTRPM8–PIP_2_ models, together with the binding data showing that hPIRT binds to the hTRPM8-S1S4 domain and PIP_2_, suggest that PIRT likely binds TRPM8 near the PIP_2_ binding site. Collectively, the data imply that PIRT interacts with TRPM8 on the S1/S4 helix face of the S1S4 domain (Fig. 5c), which would place PIRT near the TRPM8 pore domain and the TRP helix (Fig. 5d), which are emerging as critical regions related to TRP channel function and PIRT regulation^12^.

PIP_2_ plays a multitude of roles in biology^53^, and many regulatory pathways control its availability. Some of these pathways include regulation of PIP_2_ biosynthesis, interconversion between phosphoinositide species, and degradation (e.g., phospholipase C cleavage of PIP_2_ to inositol 1,4,5 tris-phosphate (IP_3_) and diacylglycerol (DAG)). Additionally, local PIP_2_ concentrations can be controlled by sequestration by PIP_2_ binding proteins^54,55^. For example, MARCKS (*M*yristoylated *a*lanine-*r*ich *C*-*k*inase *s*ubstrate) is a PIP_2_ binding protein that is thought to participate in regulating the concentration of free PIP_2_ in the membrane^55,56^. The MARCKS–PIP_2_ interaction is reversible such that free PIP_2_ concentrations are dynamically regulated^55^. PIRT, like MARCKS and other PIP_2_ binding proteins, has a polybasic region that is an electrostatic complement to the PIP_2_ head group (Fig. 2c). By analogy, PIRT regulation of TRPM8 activity, and generally for other PIRT-sensitive ion channels, may arise from the ability of PIRT to shuttle and/or sequester PIP_2_ leading to activation or attenuation respectively.

Access to PIP_2_ has strong modulatory effects on many ion channels, including many TRP channels^9,11,57–64^, with recent studies suggesting channel regulation can be controlled by access to PIP_2_^9,62–64^. The voltage-gated potassium channel KCNQ1, which is structurally homologous to hTRPM8, also requires PIP_2_ to function, where PIP_2_ is thought to stabilize the channel in an open state by maintaining coupling between the voltage-sensing domain (helices S1-S4) and the pore domain (S5-S6)^11,65^. KCNQ1 functional studies indicate that positively charged residues in the post S6 helical region are essential to PIP_2_ regulation^66^ and this region is structurally homologous to the TRP domain in hTRPM8 identified by Rohacs and coworkers to be important in PIP_2_ regulation of hTRPM8^9^. Interestingly, the KCNQ1–KCNE1 complex is also regulated by PIP_2_ where the complex function is dependent on the lipid^65,66^. Analogous to PIRT, the modulatory KCNE1 protein has a polybasic region near the inner membrane leaflet. In KCNE1 and other KCNE family members, these basic residues are key determinants of PIP_2_ sensitivity for the KCNQ1–KCNE1 complex^67^. This general mode of PIP_2_ localizing between TRPM8 and PIRT is reminiscent of KCNQ1 and KCNE1. For PIRT modulation of TRPM8, our experimental data show that hPIRT binds both PIP_2_ and the hTRPM8-S1S4 competitively (Fig. 4). A previous study identified that PIRT modulation of TRPM8 also depends on the pore domain^12^. The PIP_2_–hTRPM8 docking studies, and recent cryo-EM structural studies^50^, identify that PIP_2_ binds near the domains in TRPM8 implicated in PIRT binding and functional studies^12^. Together, these hTRPM8 regions are located in relative proximity and are consistent with a competitive model of hPIRT regulation of hTRPM8. In general, PIRT–PIP_2_ interactions could lead to increased or decreased local effective PIP_2_ concentrations, i.e., a shuttling/sequestering mechanism, that could lead to both positive and negative regulation of a given PIP_2_-dependent channel.

Our proposed mechanism for PIRT function integrates previous experimental observations that PIP_2_ depletion from TRPM8 leads to inactive TRPM8-dependent conductance^9,10,68^, that PIRT effectively desensitizes TRPM8 conductance in electrophysiological studies using human constructs^12^, that our competitive binding data shows PIRT binds TRPM8 and PIP_2_, and our structurally validated^50^ computational docking models highlight the putative PIP_2_ binding region. Our data suggest a mechanism for PIP_2_ modulation by dynamic allocation between PIRT, a PIP_2_ binding protein, and TRPM8, a PIP_2_ sensitive ion channel.

## Methods

### Purification and solubilization

Expression and purification of hPIRT follows the protocol outlined in Hilton, *et al*.^12^. The resulting hPIRT was greater than 95% pure as verified by SDS-PAGE (Supplementary Fig. S1). Following ion exchange chromatography, the buffer was exchanged and concentrated to NMR buffer (4% D_2_O (v/v), 20 mM sodium phosphate (Fisher Scientific) and 0.2 mM EDTA at pH 6.5) and a final volume of 180 μL for NMR studies. The identity of the protein was verified by LC-MS/MS (MS Bioworks) after trypsin digestion with 62% coverage of hPIRT, including regions spanning both N- and C-termini, indicating full-length protein was expressed, purified, and ultimately studied.

### Expression and purification of hTRPM8-S1S4

The expression and purification of the human TRPM8 ligand sensing domain (hTRPM8-S1S4) followed what was previously reported^12,37^.

### Far-UV circular dichroism (CD)

A Jasco J-715 spectropolarimeter was used to collect a CD spectrum of hPIRT (Supplementary Fig. S1). The CD buffer conditions were identical to those used in the NMR studies with a hPIRT concentration of 0.2 mg/mL. Temperature was maintained with a Jasco Peltier device (JASCO PTC-424S) and set to 40 °C. The data was collected from signal averaging five scans from 190 nm to 250 nm in 0.5 nm steps.

### Detergent screening hPIRT

To optimize the detergent conditions for solution NMR, hPIRT was reconstituted in six different membrane mimics and screened by ^15^N TROSY-HSQC experiments (Supplementary Fig. S2). hPIRT was expressed and purified as described above, except that Empigen was exchanged to a specific candidate detergent. The following micelle and bicelle membrane mimics were evaluated: DHPC, LMPC, DMPG:DHPC q = 0.3, LMPG, TDPC, and DPC.

### Amino acid resonance assignment and secondary structure assessment

To assign the hPIRT backbone resonances, a 0.9 mM sample of ^15^N, ^13^C labeled hPIRT in a 3 mm diameter NMR tube with 4% D_2_O (v/v) was used to collect transverse relaxation optimized spectroscopy (TROSY)^69^ versions of traditional protein backbone amide 3D experiments on a Bruker 850 MHz (^1^H) Avance III HD spectrometer with a 5 mm TCI Cryoprobe. The experiments collected include TROSY-based HSQC, HNCA, HNCOCA, HNCACB, HNCO, and CBCACONH; parameters for each experiment are listed in Supplementary Tabel S1 and representative assignment data shown in Supplementary Fig. S3. Uniformly sampled NMR experiments were processed in nmrPipe^70^ and analyzed in CcpNMR^71^. Non-uniformly sampled experiments were reconstructed using qMDD^72^, processed in NMRPipe, and analyzed in CcpNMR. The resonance assignments were deposited in the Biological Magnetic Resonance Bank (BMRB ref 27438). Human PIRT secondary structure prediction was generated from the backbone resonance assignments with the TALOS-N software^30,31^.

### Non-covalent solvent paramagnetic relaxation enhancement (PRE) studies

Resonance intensities were monitored on a 0.3 mM hPIRT sample by collecting ^1^H-^15^N TROSY-HSQC spectra at 850 MHz ^1^H with 0 mM, 2 mM, 6 mM, 10 mM, and 20 mM Gd(III)-DTPA (Santa Cruz Biotechnology) from a stock solution of 150 mM Gd(III)-DTPA in 25 mM sodium phosphate, pH 6.5 and 250 mM EDTA (Sigma Aldrich). EDTA is present to chelate any free Gd(III). The intensities of individual amino acids resonances were then plotted as a function of the concentration of added Gd(III)-DTPA and fit according to a mono-exponential decay $f(x)={e}^{-\varepsilon x}$; where ε is the solvent paramagnetic relaxation enhancement, *x* is the concentration of Gd(III)-DTPA added. The magnitude of ε was then plotted as a function of the corresponding residue number (Fig. 1d).

### ^15^N hPIRT-detected NMR titrations with hTRPM8-S1S4 and PIP_2_

^15^N-hPIRT was titrated with ^14^N-hTRPM8-S1S4 or a short chain PIP_2_ lipid, (1,2-dioctanoyl-*sn*-glycero-3-phospho-(1′-myo-inositol-4′,5′-bisphosphate).The titration with hTRPM8-S1S4:hPIRT follows what was done for Hilton, *et al*.^12^ with the exception that the mole ratios are converted to mole percentage of titrated hTRPM8-S1S4 and are 3.7 × 10^−3^, 7.5 × 10^−3^, 14.9 × 10^−3^, 29.9 × 10^−3^, 37.3 × 10^−3^, 59.8 × 10^−3^, 74.7 × 10^−3^, 112.0 × 10^−3^, 149.0 × 10^−3^, where mole percent is defined as:

$$mole\,\,percent=(\frac{{\rm{m}}{\rm{o}}{\rm{l}}.{\rm{d}}{\rm{i}}{\rm{C}}8\,{\rm{P}}{\rm{I}}(4,\,5){{\rm{P}}}_{2}}{mol.\,DPC\,+mol.\,hPIRT\,+{\rm{m}}{\rm{o}}{\rm{l}}.\,{\rm{d}}{\rm{i}}{\rm{C}}8\,{\rm{P}}{\rm{I}}(4,\,5){{\rm{P}}}_{2}\,})\times 100{\rm{ \% }}.$$

The hPIRT titration with PIP_2_ was done in a similar fashion as the hTRPM8-S1S4 domain, except that the amount of PIP_2_ used corresponds to the following mole percentages: 0.05, 0.10, 0.15, 0.20, 0.40, 0.80, and 1.5.

Changes in chemical shift were analyzed according to previously established protocols^12,38,73^. Errors in *K*_d_ were calculated using the standard deviations of the fit, by using Matlab R2017a to fit the adsorption binding model and then taking the square root along the diagonal of the covariance matrix of the fit.

### Microscale thermophoresis

Microscale thermophoresis was measured on a Nanotemper Monolith NT.115 nano Blue/Green (MO-G008) instrument. Human PIRT was labeled with the Nanotemper green maleimide reactive fluorophore. Before labeling, hPIRT was purified and solubilized in 0.1% DPC in 50 mM HEPES buffer at pH 7.5 as described above, which was then buffer exchanged to remove imidazole using a 10 kDa cutoff Amicon Ultra 5 centrifugal filter unit. After which, 100 μL of 20 μM hPIRT was reacted in the dark with 2 mM DTT for 3 h at room temperature. The fluorophore was added to a final volume of 200 μL and a concentration of 2:1 dye:protein (mol:mol). The maleimide reaction was carried out in the dark at room temperature overnight and then purified using a gravity flow desalting column. One column volume eluted the labeled protein at 6 μM concentration and a volume of 300 μL.

For the hTRPM8-S1S4 sample, 40 μL of 400 nM hPIRT was used per titration point in mole percentages of 0.1368, 0.0342, 0.0171, 0.0086, 0.0043, 0.0011, 0.0005, and 0.0001 of hTRPM8-S1S4. For the PIP_2_ sample, 40 μL of 400 nM of hPIRT was prepared with an initial 0.1 mg/mL of PIP_2_ with mole percentages of 0.421, 0.220, 0.110, 0.055, 0.028, 0.007, and 0.004. For competitive studies with saturating PIP_2,_ the hTRPM8-S1S4 was prepared as above but included 4.05 mole% PIP_2_ in each tube. All the samples were run in triplicate with 95% LED power and 40% infrared laser power. As a control, hPIRT was titrated in the same buffer conditions and protein concentration as above with DMSO concentrations ranging from 10 μM to 100 mM with no ligand-dependent thermophoresis observed (Supplementary Fig. S4). Error bars in MST measurements were calculated as fraction bound standard errors of the mean from three separate measurements.

### Rosetta comparative modeling of the human TRPM8 transmembrane domain (residues 672–1012)

A Rosetta protocol capture and PDB formatted lowest energy hTRPM8-TMD comparative model are provided in the Supplementary Materials. Briefly, RosettaCM^42^ was used to model the hTRPM8-TMD (amino acids 672–1012). Using Rosetta protocols with implicit membrane scoring functions^44–46^, 11,300 decoys were generated using *Fa*TRPM8 (6BPQ)^17^ and mTRPM4 (6BQV, 6BCL, 6BCJ, 5WP6, 6BCO)^51,52^ cryo-EM structures as templates, and the *Fa*TRPM8 EM electron density map as a restraint (EMDB: 7127). To use these structural templates, a sequence alignment was created using CLUSTAL-Ω^74^ between the hTRPM8, flycatcher TRPM8, and the mouse TRPM4 (Supplementary Fig. S6), which was manually refined to enforce a known functional disulfide bond^75^. The alignment file was then used to thread the hTRPM8 sequence onto each template. The 9mer and 3mer fragments were generated using the ROBETTA server^76^ for positions 672–1012 of the hTRPM8 amino acid sequence. The flycatcher TRPM8 cryo-EM structure has no density for the following transmembrane regions: 716–721, 820–822, 889–894, 912–945, and 978–990 and were rebuilt *de novo* in the human comparative model. All-atom refinement was carried out on the two biggest clusters identified with Calibur software^77^ comprising 379 and 241 decoys and expanded to 5600 decoys per cluster. All decoys were rescored with mpframework_fa_2007.wts^45,46^ to the respective lowest energy conformer and clustered with Calibur where the lowest energy decoy from the largest cluster was used as the representative model.

### Rosetta ligand docking of PIP_2_ to hTRPM8 transmembrane domain (residues 672–1012)

A Rosetta protocol capture for docking is provided in the Supplementary Materials. Rosetta has been used in the past to dock ligands to membrane proteins^78^, and we used a RosettaScripts^79^ protocol to computationally dock PIP_2_ (CHARMM small molecule library, CSML: SAPI24)^80^ to our hTRPM8-TMD comparative model. The initial PIP_2_ starting point was manually placed out of contact between hTRPM8-S1S4 domains with PIP_2_ in the pseudo-bilayer plane and the head group in an intracellular facing orientation (Supplementary Fig. S5). To efficiently sample PIP_2_ conformational space, 1000 PIP_2_ conformers were generated using Frog2^81^ with the AMMOS energy minimization^82^. Docking PIP_2_ to hTRPM8 followed previously optimized Rosetta protocols to generate a total of 10,000 docked hTRPM8–PIP_2_ complex decoys^79^. The Rosetta score versus the ligand PIP_2_ RMSD to the lowest energy decoy was analyzed (Supplementary Fig. S6) to assign cluster centroids and then finally each assigned centroid was individually analyzed in PyMol (Supplementary Fig. S7). The ligand RMSD corresponds to the internal symmetries and equivalent atoms within the ligand^79^. The cluster centroids were used to seed an additional 1000 Rosetta-generated decoys guided by experimental pseudo-constraints^49^ to encourage either Lys995 or Arg998 to non-covalently interact with the head group of PIP_2_. To limit computational bias, the constraint file did not direct which phosphate should interact with Lys995 or Arg998, nor did it specify whether Lys995 or Arg998 would be favored for the interaction. Separately, docking with PIP_2_ constraints guided to Arg1008 was calculated with 4000 decoys and analyzed with poor results and modes that predominantly force PIP_2_ into unlikely binding modes. It is worth noting that Arg1008 is not near enough to the positively charged pocket where Lys995 and Arg998 to satisfy one PIP_2_ molecule per monomer. Additionally, the PIP_2_ tails were loosely constrained (see PIP_2_ constraint file in Supplementary Material) by loosely enforcing the distal regions of the acyl chains to the hydrophobic core of the implicit membrane bilayer in order encourage a physiologically appropriate PIP_2_ membrane depth. After the constraint guided refinement, a small perturbation using the Transform mover was applied to increase sampling near the consensus docking site. A plot of the refined decoys score versus RMSD was analyzed with the 20 lowest energy decoys from each of three clusters were used in the analysis of hTRPM8–PIP_2_ complex and the implications for hPIRT modulation (Figs S7–S9).

## Supplementary information

hTRPM8-TMD Coordinate File 1

Supplementary Information

## Data Availability

The data generated from the current study are available from W.D.V.H. on reasonable request. hPIRT NMR assignment data have been deposited in the BMRB 27438. The lowest scoring hTRPM8-TMD decoy is included in the Supplementary Materials.

## References

[1] Proudfoot CJ (2006). Analgesia mediated by the TRPM8 cold receptor in chronic neuropathic pain. Current Biology.

[2] Tsavaler L, Shapero MH, Morkowski S, Laus R (2001). Trp-p8, a novel prostate-specific gene, is up-regulated in prostate cancer and other malignancies and shares high homology with transient receptor potential calcium channel proteins. Cancer Res.

[3] Zhang L, Barritt GJ (2004). Evidence that TRPM8 is an androgen-dependent Ca2+ channel required for the survival of prostate cancer cells. Cancer Res.

[4] Ma S (2012). Activation of the cold-sensing TRPM8 channel triggers UCP1-dependent thermogenesis and prevents obesity. Journal of molecular cell biology.

[5] Rossi HL (2012). Characterization of bilateral trigeminal constriction injury using an operant facial pain assay. Neuroscience.

[6] McKemy DD, Neuhausser WM, Julius D (2002). Identification of a cold receptor reveals a general role for TRP channels in thermosensation. Nature.

[7] Reid G, Flonta ML (2002). Ion channels activated by cold and menthol in cultured rat dorsal root ganglion neurones. Neurosci Lett.

[8] Hilton JK, Rath P, Helsell CV, Beckstein O, Van Horn WD (2015). Understanding thermosensitive transient receptor potential channels as versatile polymodal cellular sensors. Biochemistry.

[9] Rohacs T, Lopes CM, Michailidis I, Logothetis DE (2005). PI(4,5)P2 regulates the activation and desensitization of TRPM8 channels through the TRP domain. Nat Neurosci.

[10] Yudin Y, Lukacs V, Cao C, Rohacs T (2011). Decrease in phosphatidylinositol 4,5-bisphosphate levels mediates desensitization of the cold sensor TRPM8 channels. J Physiol.

[11] Liu B, Qin F (2005). Functional control of cold- and menthol-sensitive TRPM8 ion channels by phosphatidylinositol 4,5-bisphosphate. J Neurosci.

[12] Hilton JK, Salehpour T, Sisco NJ, Rath P, Van Horn WD (2018). Phosphoinositide-interacting regulator of TRP (PIRT) has opposing effects on human and mouse TRPM8 ion channels. J Biol Chem.

[13] Tang M, Wu GY, Dong XZ, Tang ZX (2016). Phosphoinositide interacting regulator of TRP (Pirt) enhances TRPM8 channel activity *in vitro* via increasing channel conductance. Acta Pharmacol Sin.

[14] Tang Z (2013). Pirt functions as an endogenous regulator of TRPM8. Nat Commun.

[15] Sarria I, Ling J, Zhu MX, Gu JG (2011). TRPM8 acute desensitization is mediated by calmodulin and requires PIP(2): distinction from tachyphylaxis. J Neurophysiol.

[16] Gkika D (2015). TRP channel-associated factors are a novel protein family that regulates TRPM8 trafficking and activity. J Cell Biol.

[17] Yin Y (2018). Structure of the cold- and menthol-sensing ion channel TRPM8. Science.

[18] Andersson DA, Chase HW, Bevan S (2004). TRPM8 activation by menthol, icilin, and cold is differentially modulated by intracellular pH. J Neurosci.

[19] Andersson DA, Nash M, Bevan S (2007). Modulation of the cold-activated channel TRPM8 by lysophospholipids and polyunsaturated fatty acids. J Neurosci.

[20] Vanden Abeele F (2006). Ca2+-independent phospholipase A2-dependent gating of TRPM8 by lysophospholipids. J Biol Chem.

[21] Morenilla-Palao C, Pertusa M, Meseguer V, Cabedo H, Viana F (2009). Lipid raft segregation modulates TRPM8 channel activity. J Biol Chem.

[22] Kim AY (2008). Pirt, a phosphoinositide-binding protein, functions as a regulatory subunit of TRPV1. Cell.

[23] Wang C (2018). Pirt Together with TRPV1 Is Involved in the Regulation of Neuropathic Pain. Neural Plast.

[24] UniProt C (2015). UniProt: a hub for protein information. Nucleic Acids Res.

[25] Patel KN, Liu Q, Meeker S, Undem BJ, Dong X (2011). Pirt, a TRPV1 modulator, is required for histamine-dependent and -independent itch. PLoS One.

[26] Wang C (2015). Pirt contributes to uterine contraction-induced pain in mice. Mol Pain.

[27] Guo W (2016). Co-localization of Pirt protein and P2X2 receptors in the mouse enteric nervous system. Purinergic Signal.

[28] Gao XF (2015). Pirt reduces bladder overactivity by inhibiting purinergic receptor P2X3. Nat Commun.

[29] Jall Sigrid, Finan Brian, Collden Gustav, Fischer Katrin, Dong Xinzhong, Tschöp Matthias H., Müller Timo D., Clemmensen Christoffer (2019). Pirt deficiency has subtle female-specific effects on energy and glucose metabolism in mice. Molecular Metabolism.

[30] Shen Y, Bax A (2015). Protein structural information derived from NMR chemical shift with the neural network program TALOS-N. Methods Mol Biol.

[31] Shen Y, Bax A (2013). Protein backbone and sidechain torsion angles predicted from NMR chemical shifts using artificial neural networks. J Biomol NMR.

[32] Beel AJ (2008). Structural studies of the transmembrane C-terminal domain of the amyloid precursor protein (APP): does APP function as a cholesterol sensor?. Biochemistry.

[33] Butterwick JA, MacKinnon R (2010). Solution structure and phospholipid interactions of the isolated voltage-sensor domain from KvAP. J Mol Biol.

[34] Jones DT (1999). Protein secondary structure prediction based on position-specific scoring matrices. J Mol Biol.

[35] Buchan DW, Minneci F, Nugent TC, Bryson K, Jones DT (2013). Scalable web services for the PSIPRED Protein Analysis Workbench. Nucleic Acids Res.

[36] Williamson MP (2013). Using chemical shift perturbation to characterise ligand binding. Progress in nuclear magnetic resonance spectroscopy.

[37] Rath P, Hilton JK, Sisco NJ, Van Horn WD (2016). Implications of Human Transient Receptor Potential Melastatin 8 (TRPM8) Channel Gating from Menthol Binding Studies of the Sensing Domain. Biochemistry.

[38] Barrett PJ (2012). The amyloid precursor protein has a flexible transmembrane domain and binds cholesterol. Science.

[39] Cheng H, Jiang X, Han X (2007). Alterations in lipid homeostasis of mouse dorsal root ganglia induced by apolipoprotein E deficiency: a shotgun lipidomics study. J Neurochem.

[40] van den Bogaart G, Meyenberg K, Diederichsen U, Jahn R (2012). Phosphatidylinositol 4,5-bisphosphate increases Ca2+ affinity of synaptotagmin-1 by 40-fold. J Biol Chem.

[41] Jerabek-Willemsen M, Wienken CJ, Braun D, Baaske P, Duhr S (2011). Molecular interaction studies using microscale thermophoresis. Assay Drug Dev Technol.

[42] Song Y (2013). High-resolution comparative modeling with RosettaCM. Structure.

[43] DiMaio F, Tyka MD, Baker ML, Chiu W, Baker D (2009). Refinement of protein structures into low-resolution density maps using rosetta. J Mol Biol.

[44] Yarov-Yarovoy V, Schonbrun J, Baker D (2006). Multipass membrane protein structure prediction using Rosetta. Proteins.

[45] Alford RF (2015). An Integrated Framework Advancing Membrane Protein Modeling and Design. PLoS Comput Biol.

[46] Barth P, Schonbrun J, Baker D (2007). Toward high-resolution prediction and design of transmembrane helical protein structures. Proc Natl Acad Sci USA.

[47] Chen VB (2010). MolProbity: all-atom structure validation for macromolecular crystallography. Acta Crystallogr D Biol Crystallogr.

[48] Barad BA (2015). EMRinger: side chain-directed model and map validation for 3D cryo-electron microscopy. Nat Methods.

[49] Richter F, Leaver-Fay A, Khare SD, Bjelic S, Baker D (2011). *De novo* enzyme design using Rosetta3. PLoS One.

[50] Yin Ying, Le Son C., Hsu Allen L., Borgnia Mario J., Yang Huanghe, Lee Seok-Yong (2019). Structural basis of cooling agent and lipid sensing by the cold-activated TRPM8 channel. Science.

[51] Autzen HE (2018). Structure of the human TRPM4 ion channel in a lipid nanodisc. Science.

[52] Guo J (2017). Structures of the calcium-activated, non-selective cation channel TRPM4. Nature.

[53] Hilgemann DW, Feng S, Nasuhoglu C (2001). The complex and intriguing lives of PIP2 with ion channels and transporters. Sci STKE.

[54] Gambhir A (2004). Electrostatic sequestration of PIP2 on phospholipid membranes by basic/aromatic regions of proteins. Biophys J.

[55] McLaughlin S, Murray D (2005). Plasma membrane phosphoinositide organization by protein electrostatics. Nature.

[56] Laux T (2000). Gap43, Marcks, and Cap23 Modulate Pi(4,5p)2 at Plasmalemmal Rafts, and Regulate Cell Cortex Actin Dynamics through a Common Mechanism. J. Cell Biol..

[57] Bandell M (2006). High-throughput random mutagenesis screen reveals TRPM8 residues specifically required for activation by menthol. Nat Neurosci.

[58] Brauchi S (2007). Dissection of the components for PIP2 activation and thermosensation in TRP channels. Proc Natl Acad Sci USA.

[59] Hansen SB, Tao X, MacKinnon R (2011). Structural basis of PIP2 activation of the classical inward rectifier K+ channel Kir2.2. Nature.

[60] Chen L (2015). Migration of PIP2 lipids on voltage-gated potassium channel surface influences channel deactivation. Sci Rep.

[61] Han B (2016). Human EAG channels are directly modulated by PIP2 as revealed by electrophysiological and optical interference investigations. Sci Rep.

[62] Sun J, MacKinnon R (2017). Cryo-EM Structure of a KCNQ1/CaM Complex Reveals Insights into Congenital Long QT Syndrome. Cell.

[63] She J (2018). Structural insights into the voltage and phospholipid activation of the mammalian TPC1 channel. Nature.

[64] Rohacs T (2014). Phosphoinositide regulation of TRP channels. Handb Exp Pharmacol.

[65] Zhang H (2003). PIP2 Activates KCNQ Channels, and Its Hydrolysis Underlies Receptor-Mediated Inhibition of M Currents. Neuron.

[66] Loussouarn G (2003). Phosphatidylinositol-4,5-bisphosphate, PIP2, controls KCNQ1/KCNE1 voltage-gated potassium channels: a functional homology between voltage-gated and inward rectifier K+ channels. EMBO J.

[67] Li Y (2011). KCNE1 enhances phosphatidylinositol 4,5-bisphosphate (PIP2) sensitivity of IKs to modulate channel activity. Proc Natl Acad Sci USA.

[68] Daniels RL, Takashima Y, McKemy DD (2009). Activity of the neuronal cold sensor TRPM8 is regulated by phospholipase C via the phospholipid phosphoinositol 4,5-bisphosphate. J Biol Chem.

[69] Czisch M, Boelens R (1998). Sensitivity enhancement in the TROSY experiment. J Magn Reson.

[70] Delaglio F (1995). NMRPipe: a multidimensional spectral processing system based on UNIX pipes. J Biomol NMR.

[71] Vranken WF (2005). The CCPN data model for NMR spectroscopy: development of a software pipeline. Proteins.

[72] Kazimierczuk K, Orekhov VY (2011). Accelerated NMR spectroscopy by using compressed sensing. Angew Chem Int Ed Engl.

[73] Kroncke BM (2016). Structural basis for KCNE3 modulation of potassium recycling in epithelia. Sci Adv.

[74] Larkin MA (2007). Clustal W and Clustal X version 2.0. Bioinformatics.

[75] Dragoni I, Guida E, McIntyre P (2006). The cold and menthol receptor TRPM8 contains a functionally important double cysteine motif. J Biol Chem.

[76] Kim DE, Chivian D, Baker D (2004). Protein structure prediction and analysis using the Robetta server. Nucleic Acids Res.

[77] Li SC, Ng YK (2010). Calibur: a tool for clustering large numbers of protein decoys. BMC Bioinformatics.

[78] Nguyen ED, Norn C, Frimurer TM, Meiler J (2013). Assessment and challenges of ligand docking into comparative models of G-protein coupled receptors. PLoS One.

[79] Lemmon G, Meiler J (2012). Rosetta Ligand docking with flexible XML protocols. Methods Mol Biol.

[80] Jo S, Kim T, Iyer VG, Im W (2008). CHARMM-GUI: a web-based graphical user interface for CHARMM. J Comput Chem.

[81] Miteva MA, Guyon F, Tuffery P (2010). Frog2: Efficient 3D conformation ensemble generator for small compounds. Nucleic Acids Res.

[82] Pencheva T, Lagorce D, Pajeva I, Villoutreix BO, Miteva MA (2008). AMMOS: Automated Molecular Mechanics Optimization tool for in silico Screening. BMC Bioinformatics.

