# Supplementary material for: Competitive Interactions between PIRT, the Cold Sensing Ion Channel TRPM8, and PIP2 Suggest a Mechanism for Regulation
Source: Sci Rep. 2019 Oct 1;9:14128. doi: 10.1038/s41598-019-49912-5 (PMC6773951; doi:10.1038/s41598-019-49912-5)
Supplement: Supplementary file 2 — Supplementary Information [file 41598_2019_49912_MOESM2_ESM.pdf]

Supplementary Materials

Competitive Interactions between PIRT, the Cold Sensing Ion Channel TRPM8, and PIP_2 Suggest a Mechanism for Regulation

Nicholas J. Sisco[1-4], Cole V. M. Helsell[1-4], Wade D. Van Horn[1-4,]*

[1]School of Molecular Sciences, Arizona State University, Tempe, AZ. 85287

[2]The Biodesign Institute, Arizona State University, Tempe, AZ. 85281

[3]The Virginia G. Piper Center for Personalized Diagnostics, Arizona State University, Tempe, AZ. 85281

[4]The Magnetic Resonance Research Center, Arizona State University, Tempe, AZ. 85287

*To whom correspondence should be addressed: Wade D. Van Horn, PO Box 871604, Tempe, AZ, USA.

Tel.: (480) 965-8322; Fax: (480) 965-2747; E-mail: wade.van.horn@asu.edu

Contents:

Table S1: NMR experimental parameters

Table S2: NMR detected binding constant table

Figure S1: SDS-PAGE, circular dichroism, and LC-MS/MS Analysis

Figure S2: Detergent optimization TROSY-HSQC spectra

Figure S3: Triple resonance assignment strip

Figure S4: Microscale thermophoresis negative control

Figure S5: Sequence alignment for Rosetta comparative modeling

Figure S6: TRPM8 transmembrane domain Rosetta comparative modeling

Figure S7: Rosetta computationally predicted PIP_2 binding site

Figure S8: PIP_2 Rosetta docking to TRPM8-TMD flow chart and energy plots

Figure S9: Unrestrained PIP_2 docking structural overlay

Figure S10: Arg1008 restrained PIP_2 docking does not show convergence

Figure S11: Analysis of recent PIP_2 bound structure vs Rosetta predicted

Rosetta protocols and protocol captures

Top scoring decoy file: hTRPM8-TMD.pdb

Experiment*	Transients	F1 (direct)		F2 (indirect)		F3 (indirect)		NUS %
		Points	SW (Hz)	Points	SW (Hz)	Points	SW (Hz)	
**^{15}N, ^{1}H HSQC**	128	2048	11029.4	128	2843.5	N/A	N/A	0
**HNCA**	64	2048	10638.3	64	5559.1	128	2585.0	50
**HN(CO)CA**	64	2048	10638.3	64	5559.1	128	2585.0	50
**HNCACB**	64	2048	10638.3	64	16039.1	128	2585.0	50
**CBCA(CO)NH**	64	2048	10638.3	64	16039.1	128	2585.0	50
**HNCO**	24	2048	10638.3	64	5559.1	128	2585.0	50

All experiments were TROSY-based

Table S1. NMR experimental parameters. NUS stands for non-uniform sampling.

NMR Binding Data				
**Residue**	**PIP$_2$ (mol%)**		**TRPM8-S1S4 (mol%)**	
**106**	**-**	**-**	**0.0173**	**0.004**
**110**	**0.14**	**0.04**	**-**	**-**
**119**	**0.26**	**0.06**	**0.0021**	**0.0004**
**120**	**0.90**	**0.20**	**0.0183**	**0.009**
**123**	**0.37**	**0.08**	**-**	**-**
**124**	**0.78**	**0.12**	**0.0062**	**0.0015**
**126**	**0.26**	**0.20**	**-**	**-**
**128**	**0.04**	**0.15**	**-**	**-**
**131**	**0.39**	**0.08**	**0.0011**	**0.0003**
**132**	**-**	**-**	**0.0182**	**0.0046**
**133**	**0.51**	**0.08**	**0.0061**	**0.0007**
**134**	**-**	**-**	**0.0203**	**0.0011**
**135**	**0.65**	**0.23**	**0.0121**	**0.0053**
**136**	**-**	**-**	**-**	**-**
**137**	**1.01**	**0.08**	**0.0032**	**0.0007**

Table S2. NMR detected binding constant table. The binding constants are reported in mole% derived from fitting chemical shift perturbations as a function of mole% concentrations of PIP$_2$ and TRPM8-S1S4 to a standard 1:1 binding isotherm. The hyphen means no observed binding.

Figure S1

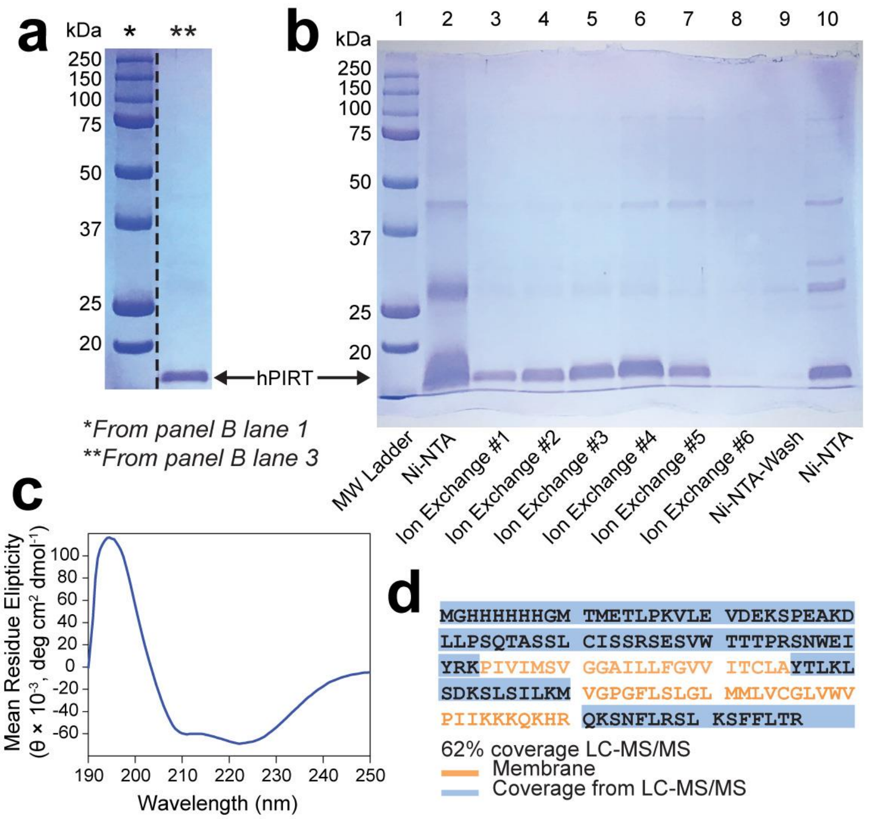

Figure S1. Purified human PIRT characterization by SDS-PAGE, far-UV circular dichroism, and mass spectrometry. (a) Cropped lanes from different parts of the same 12% SDS-PAGE gel show that hPIRT is sufficiently pure after Ni-NTA and subsequent ion exchange chromatography purification for biophysical and structural studies. These lanes are taken from lanes one and three from the full SDS-PAGE gel shown in panel b. (b) The full gel that was used to make panel a (lanes one and three). This gel also shows the purity of hPIRT after the initial Ni-NTA purification (lanes two and ten). Early ion exchange fractions were combined for the NMR and MST studies detailed in the main manuscript. In (c), circular dichroism of hPIRT at 40 °C (where all NMR experiments were carried out) show a spectrum with mean residue ellipticity minima at 210 nm and 222 nm as well as a mean residue ellipticity maximum at 194 nm characteristic of an α-helical protein. (d) Liquid chromatography-tandem mass spectrometry (LC-MS/MS) after trypsin digestion was used to validate the identity of PIRT. Regions highlighted in blue were positively identified by LC-MS/MS. The hPIRT transmembrane helices are shown in orange lettering.

Figure S2

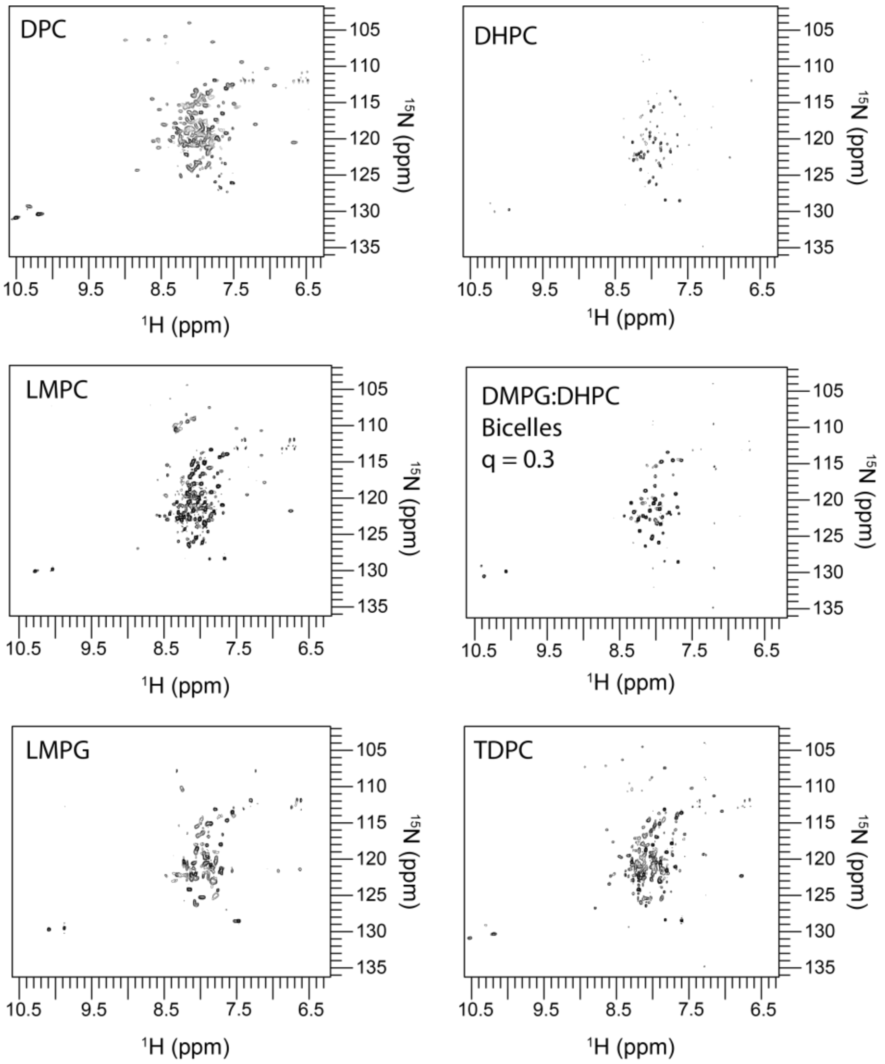

Figure S2. Membrane reconstitution detergent screens monitored with two dimensional NMR experiments. ^{1}H, ^{15}N TROSY–HSQC spectra of hPIRT at 40 °C are displayed. Based on spectral features DPC was chosen as the membrane mimic to use in pursuing structural characterization with NMR. The hPIRT spectrum reconstituted in DPC shows the narrowest line widths as well as the appropriate number of resonances correlated to the amide backbone of hPIRT as well as showing the characteristic downfield indole amine resonances from the tryptophan side chain. hPIRT has three tryptophan residues.

Figure S3

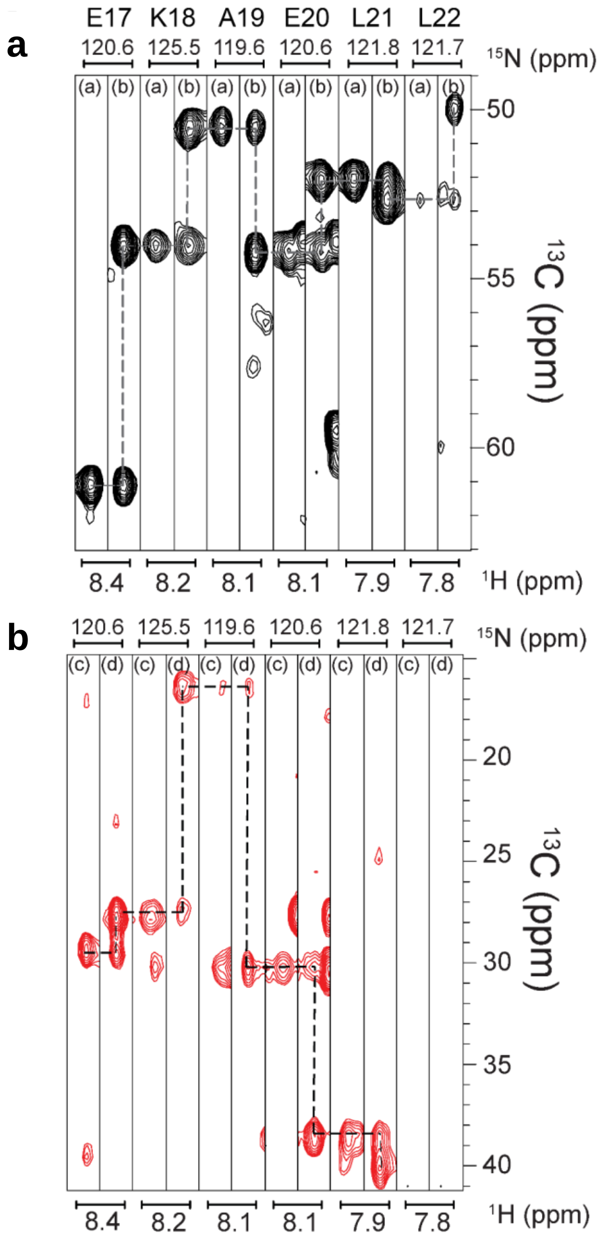

Figure S3. Representative triple resonance strip plots showing the connectivity of amino acid resonances. Panel (a) is through-bond ^{1}H-^{15}N-^{13}C C$_\alpha$ resonance correlations and (b) are ^{1}H-^{15}N-^{13}C C$_\beta$ resonance correlations from TROSY-detected HN(CO)CA and HNCA (top) or TROSY-detected CBCA(CO)NH and HNCACB data.

Figure S4

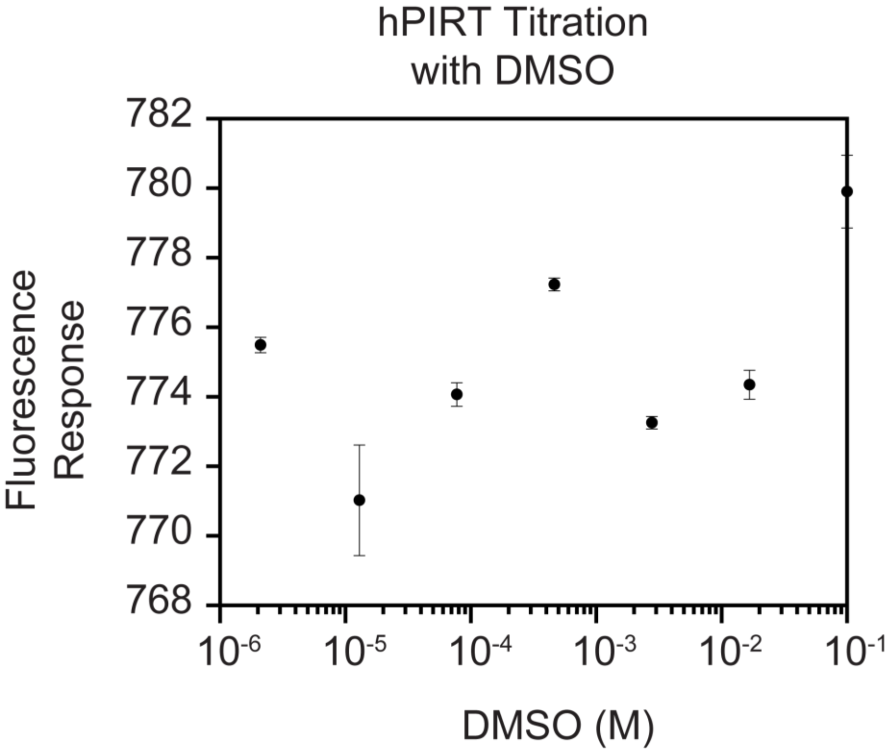

Figure S4. DMSO does not bind to PIRT. DMSO was used as a binding negative control for microscale thermophoresis. The conditions for this titration were matched to those used in the MST experiments used for PIP_2 and hTRPM8-S1S4 (see Experimental Methods). The lack of thermophoresis in a ligand-dependent manner indicates that hPIRT does not bind to DMSO.

Figure S5

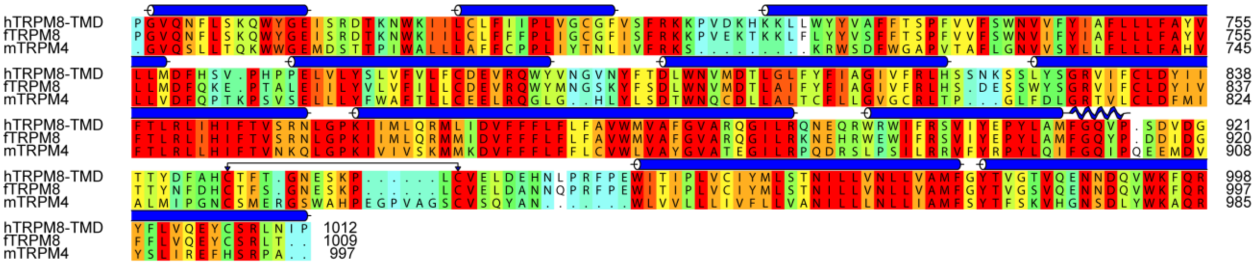

Figure S5. The alignment of hTRPM8 to the sequence of fTRPM8 (PDB: 6BPQ) and mTRPM4 (PDB: 6BQV, 6BCL, 6BCJ, 5WP6, 6BCO) that were used as a template for the homology model generated in this study. Highlighted in blue cylinders are the α-helical section, in blue squiggly is the selectivity filter, and with arrow pointed down is the disulfide that was enforced.

Figure S6

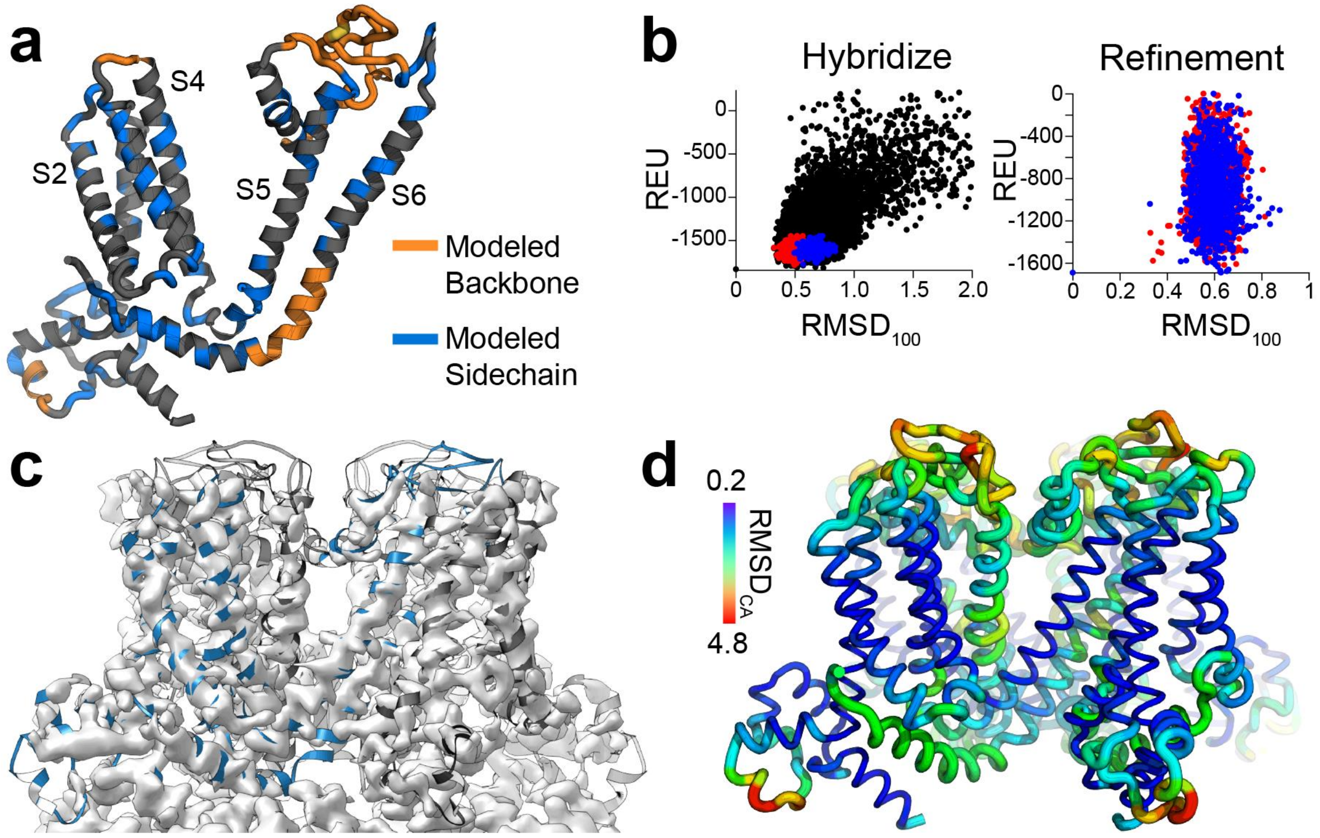

Figure S6. Comparative modeling of the human TRPM8 transmembrane domain. The collared flycatcher avian TRPM8 cryo-EM structure was used as a template with Rosetta Comparative Modeling protocols and refined with the electron density map from *Fa*TRPM8 to generate hTRPM8 TMD comparative models. (a) A representative monomer from the hTRPM8 transmembrane domain model. Orange regions were built *de novo* with RosettaCM because they were unresolved in the *Fa*TRPM8 structure. Blue regions indicate sidechains built into the comparative model that were lacking in the *Fa*TRPM8 structure. (b) RosettaCM protocols were used to model hTRPM8 showing a characteristic energy funnel with the largest clusters highlighted in red and blue. A final refinement was carried out using the EM density map as a restraint in Rosetta. (c) The hTRPM8 model tetramer fits well in the cryo-EM density map with an EM Ringer score of 1.6 indicating the model agrees with the experimental map. (d) Putty representation of the Cα RMSD magnitudes for the top ten hTRPM8-TMD models where the loop thickness and color reflect the RMSD.

Figure S7

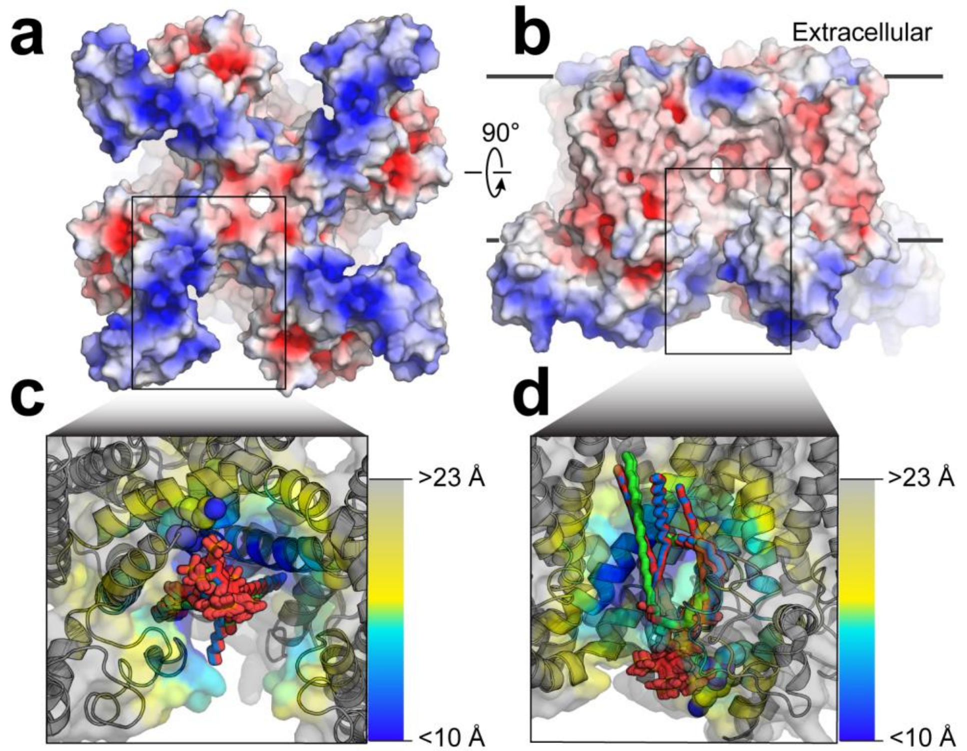

Figure S7. Rosetta predicted PIP_2 binding site in TRPM8. (a) The electrostatic surface of the TRPM8-TMD shows a generally positively charged (blue) intracellular region focused near the TRP domain and Pre-S1 helices. (b) A 90° rotation of (a) shows the electrostatic surface from the membrane plane. (c, d) Rosetta-based algorithms and experimental-based restraints were used to dock PIP_2 computationally. The docking converges to a single binding site and the best scoring PIP_2-TRPM8 complexes overlaid from the top three cluster centers, showing 60 total PIP_2 conformers.

Figure S8

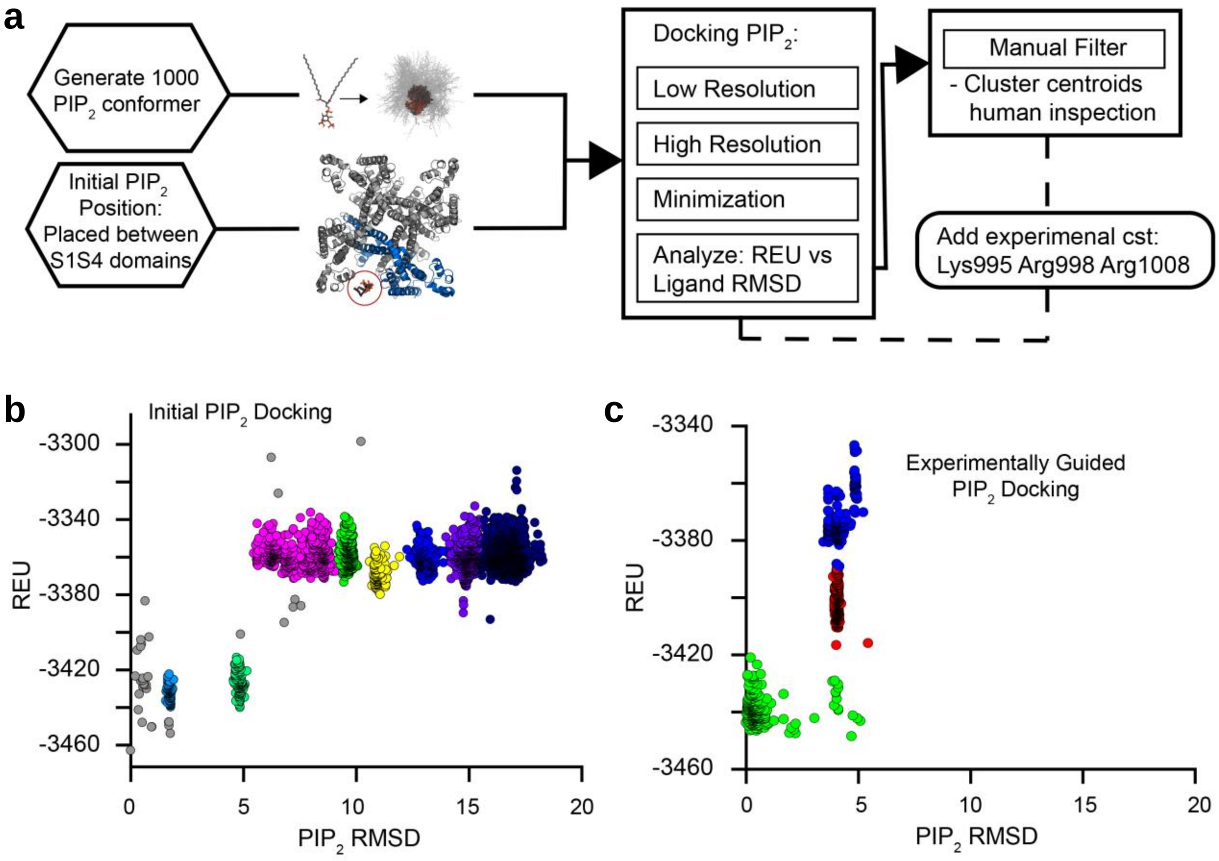

Figure S8. The general flow of Rosetta docking and score plots. (a) PIP_2 parameters are generated from 1000 conformers, and the initial starting position placed within a 10 Å radius from any contact with the TRPM8-TMD**.** Docking is carried out with 10,000 decoys of unrestrained Rosetta ligand docking in order to probe binding sites in the tetramer. The score vs. RMSD (b) shows large coverage of PIP_2 conformational space, from which the centroids of the separate clusters (colored by cluster) were analyzed in PyMol. The next round of docking used the first round centroids as seed and enforced experimental evidence of PIP_2 sensitive amino acids to guide and refine the docking. In (c), the score vs. RMSD shows well converged clusters, from which 20 from each cluster were analyzed in PyMol and used as the TRPM8–PIP_2 docked model.

Figure S9

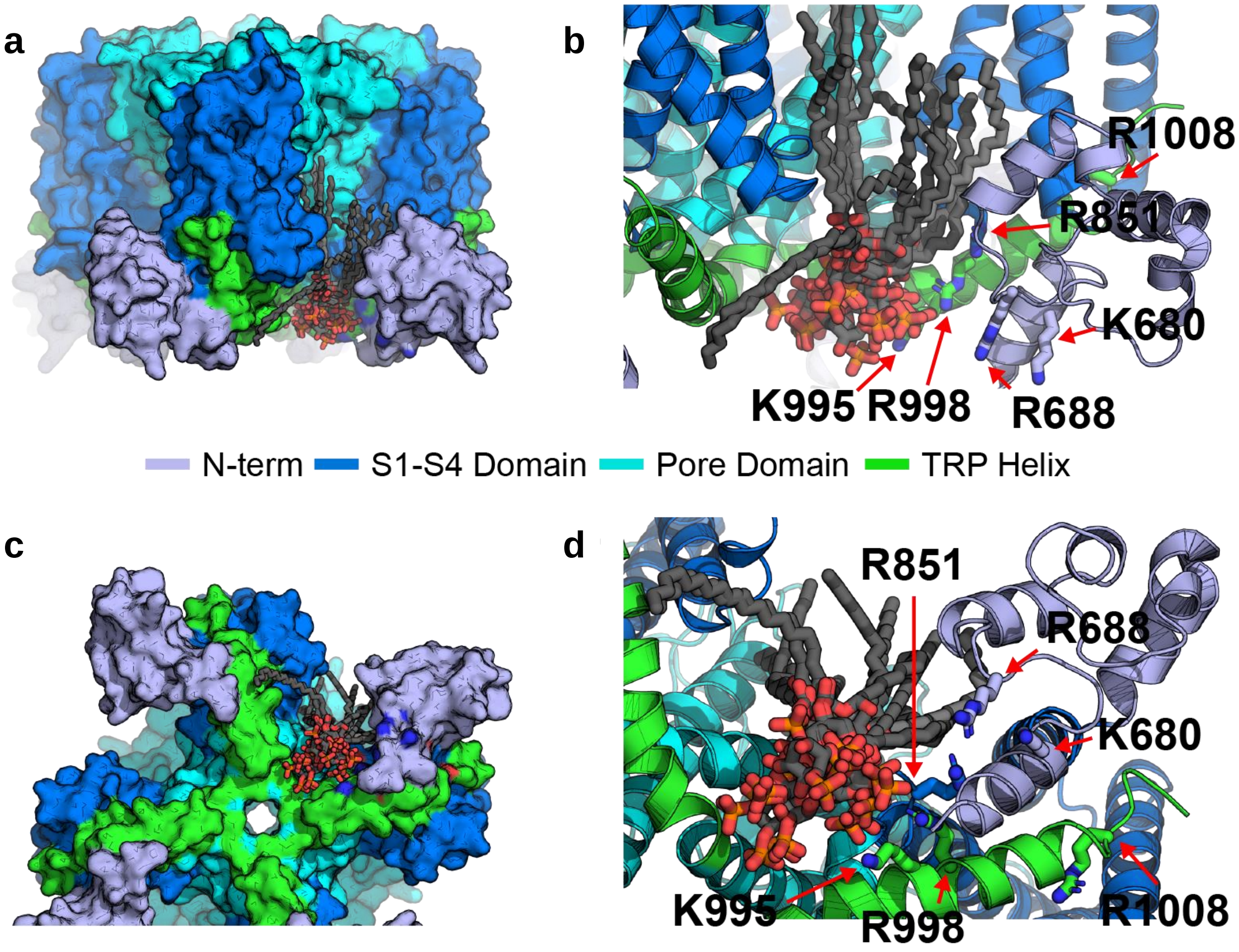

Figure S9. Using Rosetta to perform unrestrained PIP_2 docking to the human TRPM8-TMD revealed a basic pocket on the inner membrane bilayer consistent with the overall charge of this area and TRPM8 residues sensitive to PIP_2. In (a), the human TRPM8-TMD can be seen in the membrane plane colored according to: pre-S1S4 domain (purple, residues 672 to 732), S1S4 domain (blue, residues 732 to 854), pore domain (cyan, 855 to 981), and TRP domain (green, 981 to 1012). PIP_2 cluster centers overlaid and shown as sticks from the unrestrained docking calculation shown in Supplementary Figure S5B. Zooming in on the PIP_2 docking pocket in (b), basic amino acids highlighted are shown as sticks and labeled according to their identity. In (c), the human TRPM8-TMD is rotated 90° to show the intracellular facing side of the channel. Similar to (b), (d) is a zoomed in region showing the intracellular side of the docking pocket with amino acids labeled according to identity. PIP_2 can be seen adopting many conformations; however, the headgroups appear to prefer the area near Arg851, Lys995, and Arg998.

Figure S10

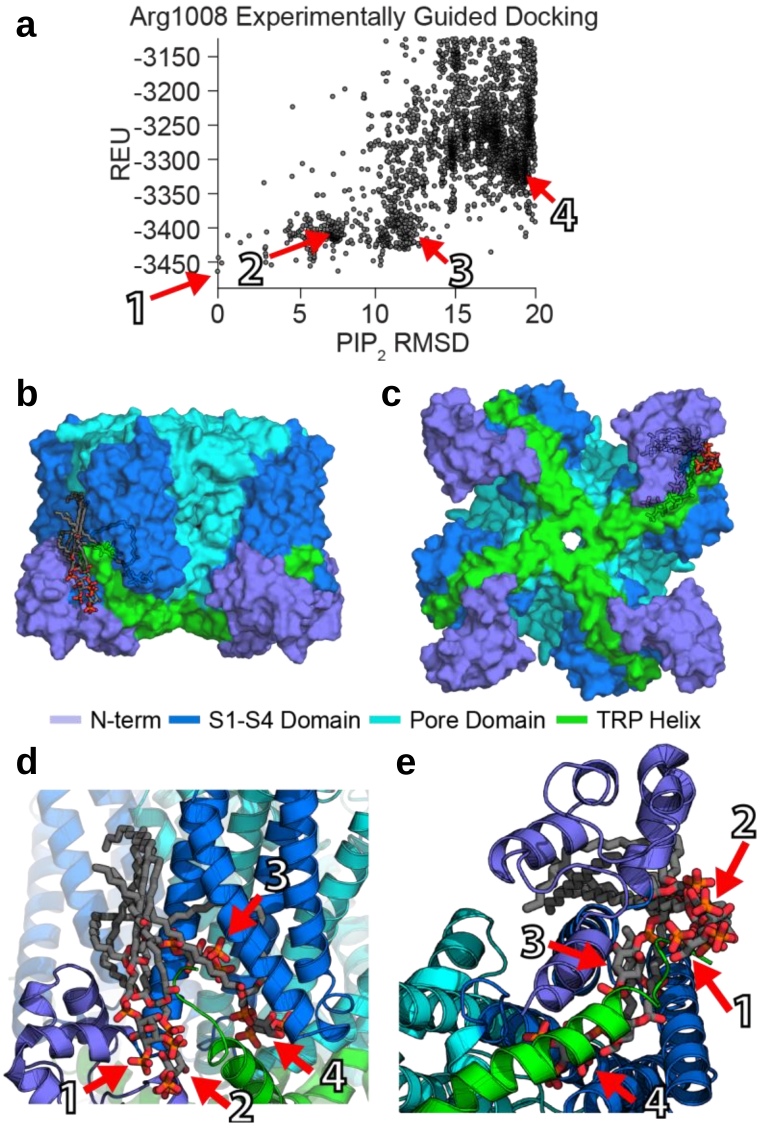

Figure S10. Experimentally guided docking for Arg1008 does not appear to converge after 4× as many decoys as experimentally refined Lys995 and Arg998. In (a), the score vs. PIP_2 RMSD shows no apparent clusters; however, manually selecting the lowest energy (1), apparent clusters 2, 3, and 4 is shown by arrows. The cluster pocket is shown in (b) and (c) with PIP_2 shown as sticks and colored as described in Supplementary Figure S6. Zooming into where PIP_2 was docked in (d) and (e), the labeled PIP_2 molecules reflect the labeling in (a), from which PIP_2 does not adopt a conserved conformation even after more sampling. These results suggest that our computational docking was not able to capture conformation to help explain Arg1008 sensitivity to PIP_2. This result does not rule out PIP_2 binding.

Figure S11

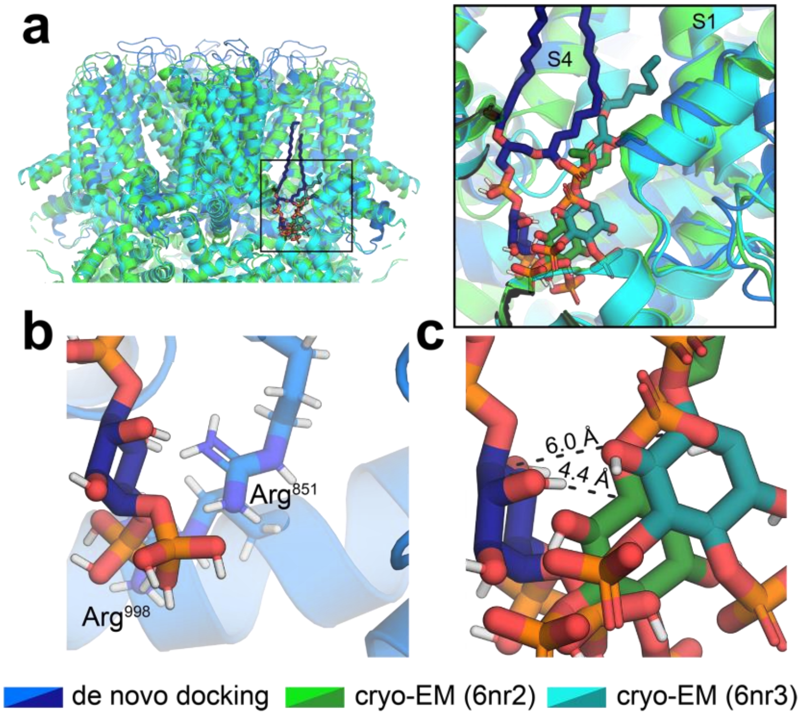

Figure S11. Comparison of PIP_2 binding sites between computationally predicted hTRPM8-TMD and *Fa*TRPM8 cryo-EM structures. The recent PIP_2 bound structures of *Fa*TRPM8 are consistent with and validate the experimentally guided Rosetta docking outlined in this manuscript. a) The overlay of the hTRPM8-TMD model docked to PIP_2 (Blue), with the cryo-EM structures of avian TRPM8-PIP_2 (Cyan and Green). Inset right is a close up of the similarities between the computationally predicted and structurally determined PIP_2 binding sites. The human TRPM8 model and PIP_2 bound structures are similar with an Cα RMSD between 1.1 to 1.9 Å to the apo *Fa*TRPM8 structure (6bpq). b) Arginine residues validated structurally and functionally to be important to PIP_2 binding are highlighted in the computational hTRPM8–PIP_2 complex. c) The distances between the C1 carbons of PIP_2 from the computationally identified and structurally determined binding sites are relatively close in space and vary between 4.4 Å (6nr2) to 6.0 Å (6nr3). We note that the reported resolutions of these structures are 4.0 Å and 3.4 Å for 6nr2 and 6nr3, respectively.

ROSETTA INPUT FILES AND COMMANDS

Rosetta was compiled using Rosetta release 3.8 with GNU compiler collection (gcc) and message passing interface (mpi) on CenOS 7 Linux distribution. The calculations were run on a combination of Intel Xeon Broadwell processors in the Van Horn lab or at the ASU Research Computing facility.

*The numbering for the comparative modeling is based on a starting amino acid P672 renamed to P1 for convenience in the following files and was later renumbered to P672.

-TRPM8 TM Span File-

```
TM region prediction
8 341
antiparallel
n2c
 23  35   23  35
 65  86   65  86
 94 117   94 117
120 145  120 145
157 179  157 179
190 215  190 215
233 244  233 244
285 310  285 310
```

-TRPM8 Disulfide Bond-

```
258 269
```

-Example Grishin Alignment FIle-

Alignment of hTRPM8-TMD to FaTRPM8.

```
## hTRPM8.pdb 6bpq.pdb
#
scores from program: 0
0
PGVQNFLSKQWYGEISRDTKNWKIILCLFIIPLVGCGFVSFRKKPVDKHKKLLWYYVAFFTSPFVVFSWNVVFYIA
FLLLFAYVLLMDFHSV-
PHPPELVLYSLVFVLFCDEVRQWYVNGVNYFTDLWNVMDTLGLFYFIAGIVFRLHSSNKSSLYSGRVIFCLDYIIF
TLRLIHIFTVSRNLGPKIIMLQRMLIDVFFFLFLFAVWMVAFGVARQGILRQNEQRWRWIFRSVIYEPYLAMFGQV
P-SDVDGTTYDFAHCTFT-GNESKP------
LCVELDEHNLPRFPEWITIPLVCIYMLSTNILLVNLLVAMFGYTVGTVQENNDQVWKFQRYFLVQEYCSRLNIP
0
PGVQNFLSKQWYGEISRDTKNWKIILCLFFFPLIGCGFISFRKKPVEKTKKLFLYYVSFFTSPFVVFSWNVIFYIA
FLLLFAYVLLMDFQKE-PTALEIILYVLVFILLCDEVRQWYMNGSKYFSDLWNVMDTLAIFYFIAGIVFRLHS-
DESSWYSGRVIFCLDYIVFTLRLIHIFTVSRNLGPKIIMLQRMMIDVFFFLFLFAVWMVAFGVARQGILRKNEHRW
EWIFRSVIYEPYLAMFGQYP-DDIDGTTYNFDHCTFS-GNESKP------
LCVELDANNQPRFPEWITIPLVCIYMLSTNILLVNLLVAMFGYTVGSVQENNDQVWKFQRFFLVQEYCSRLT--
```

-Threading-

```
$ROSETTA/bin/partial_thread.default.linuxgccrelease -in:file:fasta hTRPM8_TMD.fasta
-in:file:alignment hTRPM8_6bqv.grishin -in:file:template_pdb 6bqv.pdb

$ROSETTA/bin/partial_thread.default.linuxgccrelease -in:file:fasta hTRPM8_TMD.fasta
-in:file:alignment hTRPM8_6bpq.grishin -in:file:template_pdb 6bpq.pdb

$ROSETTA/bin/partial_thread.default.linuxgccrelease -in:file:fasta hTRPM8_TMD.fasta
-in:file:alignment hTRPM8_6bcj.grishin -in:file:template_pdb 6bcj.pdb

$ROSETTA/bin/partial_thread.default.linuxgccrelease -in:file:fasta hTRPM8_TMD.fasta
-in:file:alignment hTRPM8_6bco.grishin -in:file:template_pdb 6bco.pdb

$ROSETTA/bin/partial_thread.default.linuxgccrelease -in:file:fasta hTRPM8_TMD.fasta
-in:file:alignment hTRPM8_6bcl.grishin -in:file:template_pdb 6bcl.pdb

$ROSETTA/bin/partial_thread.default.linuxgccrelease -in:file:fasta hTRPM8_TMD.fasta
-in:file:alignment hTRPM8_5wp6.grishin -in:file:template_pdb 5wp6.pdb
```

-Creating Symmetric Definition for hTRPM8-TMD-

```
$ROSETTAHOME/source/src/apps/public/symmetry/make_symmdef_file.pl -m NCS -a A -i B
-p 6bpq_TMD.pdb -r 1000.0 > TRPM8.symm
```

*where the TMD is the tetramer with residues 672-1012

-Experimental Restraint file for Docking-

```
# Block 1: for Lys or Arg to any Phosphate in PIP2
CST::BEGIN
  TEMPLATE::   ATOM_MAP: 1 atom_type: Phos
  TEMPLATE::   ATOM_MAP: 1 residue3: PIP

  TEMPLATE::   ATOM_MAP: 2 atom_type: Hpol ,
  TEMPLATE::   ATOM_MAP: 2 residue1:  KR
  CONSTRAINT:: distanceAB:   2.00  0.50 100.  0
CST::END
# loosely restraining a hydrogen bond distance
# These are to guide docking. The next option in
# the Rosetta Script is unrestrained perturbations.
# CONSTRAINT:: distance in A, VALUE, SD, Harmonic Weight, 0 = not covalent
# block 2 Constraint to enforce PIP2 depth by restraining
# distal PIP2 carbons in one hydrophobic tail to the middle of
# membrane


# Block 2: Constraint to enforce PIP2 depth by restraining
## distal PIP2 carbons in other hydrophobic tail to the middle of
## membrane
CST::BEGIN
  TEMPLATE::   ATOM_MAP: 1 atom_name: C15 C14 C16
  TEMPLATE::   ATOM_MAP: 1 residue3: PIP

  TEMPLATE::   ATOM_MAP: 2 atom_type: Hapol ,
  TEMPLATE::   ATOM_MAP: 2 residue1:  VA

  CONSTRAINT:: distanceAB:   5.00 2.00 100.  0
```

```
CST::END
# Block 3: Constraint to enforce PIP2 depth by restraining
## distal PIP2 carbons in other hydrophobic tail to the middle of
## membrane
CST::BEGIN
  TEMPLATE::   ATOM_MAP: 1 atom_name: C39 C38 C37
  TEMPLATE::   ATOM_MAP: 1 residue3: PIP

  TEMPLATE::   ATOM_MAP: 2 atom_type: Hapol ,
  TEMPLATE::   ATOM_MAP: 2 residue1:  VA

  CONSTRAINT:: distanceAB:   5.00  2.0 100.  0
#loosely restrained harmonic with large SD.

CST::END
```

-Rosetta CM-

```
# i/o
#
# Assuming Rosetta database path is set as $ROSETTA3_DB is set to
# $ROSETTAHOME/database
#
# Assuming Rosetta was compiled using gcc with MPI extras with
#
# scons -j NP mode=release extras=mpi bin; where NP is number of
# processors available.
#
# Command line to run this script is
#
# mpiexec -n NP rosetta_scripts.mpi.linuxgccrelease @rosetta_cm.options
#


-in:file:fasta fasta/hTRPM8_TMD.fasta
-parser:protocol rosetta_cml.xml

###########################
-nstruct 10000
-default_max_cycles 200 #
-out:file:scorefile 20180303_hTRPM8.fsc
-out:file:silent 20180303_hTRPM8.out
-out:file:silent_struct_type binary
#-out:output
-out::suffix _agave_v4_20180302_3
############################

-ignore_unrecognized_res
# relax options
-relax:minimize_bond_angles
-relax:minimize_bond_lengths
-relax:jump_move true
-relax:min_type lbfgs_armijo_nonmonotone
-relax:jump_move true
#-relax:default_repeats 2
-score:weights stage3_rlx_membrane.wts
-use_bicubic_interpolation
-hybridize:stage1_probability 1.0
# EM Density
-edensity::mapfile em_map/emd_7127.map
-edensity::cryoem_scatterers
-edensity::mapreso 4.1
-edensity::grid_spacing 1.3
#
# reduce memory footprint
-chemical:exclude_patches LowerDNA  UpperDNA Cterm_amidation SpecialRotamer
VirtualBB ShoveBB VirtualDNAPhosphate VirtualNTerm CTermConnect sc_orbitals
pro_hydroxylated_case1 pro_hydroxylated_case2 ser_phosphorylated thr_phosphorylated
tyr_phosphorylated tyr_sulfated lys_dimethylated lys_monomethylated
lys_trimethylated lys_acetylated glu_carboxylated cys_acetylated tyr_diiodinated
N_acetylated C_methylamidated MethylatedProteinCterm
#Initialize membrane
-membrane
-in:file:spanfile ./span/hTRPM8_TMD.span
```

```
-membrane:no_interpolate_Mpair
-membrane:Menv_penalties
```

-Rosetta_cml.xml-

```
<ROSETTASCRIPTS>
    <TASKOPERATIONS>
    </TASKOPERATIONS>
    <SCOREFXNS>
        <ScoreFunction name="stage1" weights="stage1_membrane.wts" symmetric="1">
            <Reweight scoretype="atom_pair_constraint" weight="0.25"/>
            <Reweight scoretype="elec_dens_fast" weight="10"/>
        </ScoreFunction>
        <ScoreFunction name="stage2" weights="stage2_membrane.wts" symmetric="1">
            <Reweight scoretype="atom_pair_constraint" weight="0.25"/>
            <Reweight scoretype="elec_dens_fast" weight="10"/>
        </ScoreFunction>
        <ScoreFunction name="fullatom" weights="stage3_rlx_membrane.wts"
symmetric="1">
            <Reweight scoretype="atom_pair_constraint" weight="0.25"/>
            <Reweight scoretype="elec_dens_fast" weight="25"/>
        </ScoreFunction>
    </SCOREFXNS>
    <FILTERS>
    </FILTERS>
    <MOVERS>
        <Hybridize name="hybridize" stage1_scorefxn="stage1"
stage2_scorefxn="stage2" fa_scorefxn="fullatom" batch="1"
stage1_increase_cycles="1.0" stage2_increase_cycles="1.0" linmin_only="0"
realign_domains="0">
            <Fragments three_mers="./frags/hTRPM8_TMD_03_05.200_v1_3"
nine_mers="./frags/hTRPM8_TMD_09_05.200_v1_3"/>
            <Template pdb="../2_threading/threaded/hTRPM8_on_6bqv.pdb"
cst_file="AUTO" weight="1.000" symmdef="../symmetry/TRPM8.symm"/>
            <Template pdb="../2_threading/threaded/hTRPM8_on_6bcl.pdb"
cst_file="AUTO" weight="1.000" symmdef="../symmetry/TRPM8.symm"/>
            <Template pdb="../2_threading/threaded/hTRPM8_on_6bpq_v3.pdb"
cst_file="AUTO" weight="1.000" symmdef="../symmetry/TRPM8.symm"/>
            <Template pdb="../2_threading/threaded/hTRPM8_on_6bcj_v3.pdb"
cst_file="AUTO" weight="1.000" symmdef="../symmetry/TRPM8.symm"/>
            <Template pdb="../2_threading/threaded/hTRPM8_on_5wp6.pdb"
cst_file="AUTO" weight="1.000" symmdef="../symmetry/TRPM8.symm"/>
            <Template pdb="../2_threading/threaded/hTRPM8_on_6bco.pdb"
cst_file="AUTO" weight="1.000" symmdef="../symmetry/TRPM8.symm"/>
            <DetailedControls start_res="289" stop_res="294" sample_template="0"
sample_abinitio="1"/>
        </Hybridize>
    </MOVERS>
    <APPLY_TO_POSE>
    </APPLY_TO_POSE>
    <PROTOCOLS>
        <Add mover="hybridize"/>
    </PROTOCOLS>

</ROSETTASCRIPTS>
```

-Refinement Options-

```
#i/o
#
# Assuming Rosetta database path is set as $ROSETTA3_DB is set to
# $ROSETTAHOME/database
#
# Assuming Rosetta was compiled using gcc and MPI extras with
#
# scons -j NP mode=release extras=mpi bin; where NP is number of
# processors available.
#
# Command line to run this script is
#
# mpiexec -n NP relax.mpi.linuxgccrelease @rosetta_cm.options
#
#
#

#### User Input #####
-in:file:l clean_list
-in::file::spanfile ../../3_hybridize/span/hTRPM8_TMD.span #read predicted
transmembrane regions
-out:suffix _relax_em_cluster1
-out:file:silent 20180307_relax_cluter1.out
-out:file:scorefile cluster1_score_raw.out
-symmetry:symmetry_definition ../../symmetry/TRPM8_em.symm
-nstruct 1
####################
#
-in:fix_disulf ../disulf.cst #read disulfide connectivity information
-detect_disulf true
-detect_disulf_tolerance 2
-ex1
-ex2
-membrane:no_interpolate_Mpair # membrane scoring specification
-membrane:Menv_penalties       # turn on membrane penalty scores
-score:weights membrane_highres_Menv_smooth.wts
-relax:thorough
-relax:dualspace               #use dualspace relax protocol
-relax:minimize_bond_angles    #dualspace relax protocol setting
-set_weights cart_bonded .5 pro_close 0 #score proline ring closure using energy
term for all bond lengths (pro_close uses virtual atom NV for proline ring scores)
-default_max_cycles 200

# EM Density
-edensity::mapfile ../../3_hybridize/em_map/emd_7127.map
-edensity::cryoem_scatterers
-edensity::fastdens_wt 25.0
-edensity::mapreso 4.1
-edensity::grid_spacing 1.3
-crystal_refine
```

-Rosetta Docking Options-

```
# Adopted from Ligand Docking from
# $ROSETTAHOME/demos/tutorials/ligand_docking/
#Pound signs indicate comments

#-in:file:s option imports the protein and ligand PDB structures
#-in:file:extra_res_fa option imports the parameters for the ligand
#
# Prepare a pdb file that has the ligand and protein in the same file.
-in
      -file
            -s M8_tet_pip2_1_ignorechain.pdb # PIP2 is chain X
            -extra_res_fa PIP.params #location of PIP2 parameter file

# Params file generated by the following script available in Rosetta3
# $ROSETTAHOME/main/source/scripts/python/public/molfile_to_params.py -n PIP -p PIP
--conformers-in-one-file pip2.sdf

#the packing options allow Rosetta to sample additional rotamers for
#protein sidechain angles chi 1 (ex1) and chi 2 (ex2)
#no_optH false tells Rosetta to optimize hydrogen placements
#flip_HNQ tells Rosetta to consider HIS,ASN,GLN hydrogen flips
#ignore_ligand_chi prevents Roseta from adding additional ligand rotamer

-packing
      -ex1
      -ex2
      -ex1aro
      -no_optH false
      -flip_HNQ true
      -ignore_ligand_chi true


#parser:protocol locates the XML file for RosettaScripts

-parser
      -protocol dock_X.xml # change to .xml for initial or restrained

#overwrite allows Rosetta to write over previous structures and scores
-overwrite

#Ligand docking is not yet benchmarked with the updated scoring function
#This flag restores certain parameters to previously published values

-mistakes
      -restore_pre_talaris_2013_behavior true
#
-nstruct 1 # 10000 or 1000 for probe vs refine respectively
-qsar:grid_dir grid_dir/ # Make this directory before running, can speed up docking
if
                         # a grid has been used previously.
-run:preserve_header # Add this to enforce the experimental restraints in pdb
header
```

-Rosetta Initial Docking XML-

```
<ROSETTASCRIPTS>

		<SCOREFXNS>
			<ScoreFunction name="ligand_soft_rep" 
weights="ligand_soft_rep">
				Reweighted based on "An integrated framework advancing 
membrane protein modeling and designâ€□ Alford, RF, et al.
				<Reweight scoretype="fa_elec" weight="0.42"/>
				<Reweight scoretype="hbond_bb_sc" weight="2.34"/>
				<Reweight scoretype="hbond_sc" weight="2.2"/>
				<Reweight scoretype="rama" weight="0.2"/>
			</ScoreFunction>
			<ScoreFunction name="hard_rep" weights="ligand">
				<Reweight scoretype="fa_intra_rep" weight="0.004"/>
				<Reweight scoretype="fa_elec" weight="0.42"/>
				<Reweight scoretype="hbond_bb_sc" weight="2.34"/>
				<Reweight scoretype="hbond_sc" weight="2.2"/>
				<Reweight scoretype="rama" weight="0.2"/>
			</ScoreFunction>
		</SCOREFXNS>

		<LIGAND_AREAS>
			<LigandArea name="inhibitor_dock_sc" chain="X" cutoff="6.0" 
add_nbr_radius="true" all_atom_mode="false"/>
			<LigandArea name="inhibitor_final_sc" chain="X" 
cutoff="6.0" add_nbr_radius="true" all_atom_mode="true"/>
			<LigandArea name="inhibitor_final_bb" chain="X" 
cutoff="7.0" add_nbr_radius="false" all_atom_mode="true" 
Calpha_restraints="0.3"/>
		</LIGAND_AREAS>

		<INTERFACE_BUILDERS>
			<InterfaceBuilder name="side_chain_for_docking" 
ligand_areas="inhibitor_dock_sc"/>
			<InterfaceBuilder name="side_chain_for_final" 
ligand_areas="inhibitor_final_sc"/>
			<InterfaceBuilder name="backbone" 
ligand_areas="inhibitor_final_bb" extension_window="3"/>
		</INTERFACE_BUILDERS>

		<MOVEMAP_BUILDERS>
			<MoveMapBuilder name="docking" 
sc_interface="side_chain_for_docking" minimize_water="false"/>
			<MoveMapBuilder name="final" 
sc_interface="side_chain_for_final" bb_interface="backbone" 
minimize_water="false"/>
		</MOVEMAP_BUILDERS>

		<SCORINGGRIDS ligand_chain="X" width="15">
```

```
                <HbdGrid grid_name="classic" weight="1.0"/> Hydrogen bond
donating grid
        </SCORINGGRIDS>

        <MOVERS>
                <Transform name="transform" chain="X" box_size="10.0"
move_distance="0.2" angle="20" cycles="10" repeats="1" temperature="5"  />
                <HighResDocker name="high_res_docker" cycles="50"
repack_every_Nth="2" scorefxn="ligand_soft_rep" movemap_builder="docking"/>
                <FinalMinimizer name="final" scorefxn="hard_rep"
movemap_builder="final"/>
                <InterfaceScoreCalculator name="add_scores" chains="X"
scorefxn="hard_rep" native="M8_tet_pip2_1_ignorechain_0193_0001.pdb"/>
        </MOVERS>

        <PROTOCOLS>
                <Add mover_name="transform"/> Large 10 angstrom Monte Carlo
spherical perturbation
                <Add mover_name="high_res_docker"/>
                <Add mover_name="final"/>
                <Add mover_name="add_scores"/>
        </PROTOCOLS>


</ROSETTASCRIPTS>
```

-Rosetta Restrained Docking XML-

```
<ROSETTASCRIPTS>

            <SCOREFXNS>
                  <ScoreFunction name="ligand_soft_rep" weights="ligand_soft_rep">
                        Reweighted based on "An integrated framework advancing
membrane protein modeling and designâ€□ Alford, RF, et al.
                        <Reweight scoretype="fa_elec" weight="0.42"/>
                        <Reweight scoretype="hbond_bb_sc" weight="2.34"/>
                        <Reweight scoretype="hbond_sc" weight="2.2"/>
                        <Reweight scoretype="rama" weight="0.2"/>
                  </ScoreFunction>
                  <ScoreFunction name="hard_rep" weights="ligand">
                        <Reweight scoretype="fa_intra_rep" weight="0.004"/>
                        <Reweight scoretype="fa_elec" weight="0.42"/>
                        <Reweight scoretype="hbond_bb_sc" weight="2.34"/>
                        <Reweight scoretype="hbond_sc" weight="2.2"/>
                        <Reweight scoretype="rama" weight="0.2"/>
                  </ScoreFunction>
            </SCOREFXNS>

            <LIGAND_AREAS>
                  <LigandArea name="inhibitor_dock_sc" chain="X" cutoff="6.0"
add_nbr_radius="true" all_atom_mode="false"/>
                  <LigandArea name="inhibitor_final_sc" chain="X" cutoff="6.0"
add_nbr_radius="true" all_atom_mode="true"/>
                  <LigandArea name="inhibitor_final_bb" chain="X" cutoff="7.0"
add_nbr_radius="false" all_atom_mode="true" Calpha_restraints="0.3"/>
            </LIGAND_AREAS>

            <INTERFACE_BUILDERS>
                  <InterfaceBuilder name="side_chain_for_docking"
ligand_areas="inhibitor_dock_sc"/>
                  <InterfaceBuilder name="side_chain_for_final"
ligand_areas="inhibitor_final_sc"/>
                  <InterfaceBuilder name="backbone"
ligand_areas="inhibitor_final_bb" extension_window="3"/>
            </INTERFACE_BUILDERS>

            <MOVEMAP_BUILDERS>
                  <MoveMapBuilder name="docking"
sc_interface="side_chain_for_docking" minimize_water="false"/>
                  <MoveMapBuilder name="final" sc_interface="side_chain_for_final"
bb_interface="backbone" minimize_water="false"/>
            </MOVEMAP_BUILDERS>

            <SCORINGGRIDS ligand_chain="X" width="15">
                  <HbdGrid grid_name="classic" weight="1.0"/> Hydrogen bond
donating grid
            </SCORINGGRIDS>

            <MOVERS>
                  <Transform name="transform_after_cst" chain="X" box_size="1.0"
move_distance="0.2" angle="5" cycles="10" repeats="1" temperature="3"  />
                  <HighResDocker name="high_res_docker" cycles="50"
repack_every_Nth="2" scorefxn="ligand_soft_rep" movemap_builder="docking"/>
```

```
                  <FinalMinimizer name="final" scorefxn="hard_rep"
movemap_builder="final"/>
                  <InterfaceScoreCalculator name="add_scores" chains="X"
scorefxn="hard_rep" native="M8_tet_pip2_1_ignorechain_0193_0001.pdb"/>
                  <AddOrRemoveMatchCsts name="cst" cst_instruction="add_new"
cstfile="pip2_v2.cst" />
            </MOVERS>

            <PROTOCOLS>
                  Add mover_name="cst" />
                  <Add mover_name="transform_after_cst" /> small 1 angstrom Monte
Carlo spherical perturbation after experimental restraints
                  <Add mover_name="high_res_docker"/>
                  <Add mover_name="final"/>
                  <Add mover_name="add_scores"/>
            </PROTOCOLS>


</ROSETTASCRIPTS>
```
